# Can people-centered community-oriented interventions improve skilled birth attendance? Evidence from a quasi-experimental study in rural communities of Cambodia, Kenya, and Zambia

**DOI:** 10.1186/s12884-020-03223-0

**Published:** 2020-09-05

**Authors:** Anbrasi Edward, Aparna Krishnan, Grace Ettyang, Younghee Jung, Henry B. Perry, Annette E. Ghee, Jane Chege

**Affiliations:** 1 Department of International Health, Johns Hopkins Bloomberg School of Public Health, 615 N Wolfe St, Baltimore, MD 21205 USA; 2 Johns Hopkins Medical Institutions, 733 North Broadway, Baltimore, MD 21205-2196 USA; 3 Moi University School of Public Health, Eldoret, Kenya; 4 WHO Timor-Leste Office United Nations House Caicoli street, Dili, Timor-Leste; 5 Department of Global Health, University of Washington, Seattle, WA USA; 6 World Vision International, Washington DC, USA

**Keywords:** Skilled birth attendance, Community health workers, Social accountability mechanisms, Community scorecards

## Abstract

**Background:**

Skilled attendance at delivery is a key marker for reducing maternal mortality. Effective community engagement strategies complemented by community health worker (CHW) services can improve access to maternal health services in areas with limited health infrastructure or workforce.

**Methods:**

A quasi-experimental study with matched comparison groups was conducted in Cambodia, Kenya and Zambia to determine the effect of integrated community investments on skilled birth attendance (SBA). In each country, communities in two districts/sub-districts received a package of community-oriented interventions comprised of timed CHW household health promotion for maternal, newborn and child health complemented by social accountability mechanisms using community scorecards. Two matched comparison districts/sub-districts received ongoing routine interventions. Data from the final evaluation were examined to determine the effect of timed CHW services and community-oriented interventions on SBA.

**Results:**

Over 80% of the 3037 women in Cambodia, 2805 women in Kenya and 1171 women in Zambia reported SBA. Women in intervention sites who received timely CHW health promotion and social accountability mechanisms in Cambodia showed significantly higher odds of SBA (aOR = 7.48; 95% CI: 3.87, 14.5). The findings also indicated that women over the age of 24 in Cambodia, women with primary or secondary education in Cambodia and secondary education in Kenya, women from higher wealth quintiles in Cambodia, and women with four or more antenatal care (ANC) visits in all countries reported significantly higher odds of SBA. Inclusion of family members in pregnancy-related discussions in Kenya (aOR = 2.12; 95% CI: 1.06, 4.26) and Zambia (aOR = 6.78; 95% CI: 1.15, 13.9) and follow up CHW visits after a referral or health facility visit (aOR = 2.44; 95% CI: 1.30, 4.60 in Cambodia; aOR = 2.17; 95% CI 1.25, 3.75 in Kenya; aOR = 1.89; 95% CI: 1.05, 2.02 in Zambia) also showed significantly greater odds of SBA.

**Conclusions:**

Enhancing people-centered care through culturally appropriate community-oriented strategies integrating timely CHW health promotion and social accountability mechanisms shows some evidence for improving SBA during delivery. These strategies can accelerate the achievement of the sustainable development goals for maternal child and newborn health.

## Background

Recent evidence from the World Health Organization indicates that globally almost 80% of births are now assisted by skilled personnel during delivery [1]. However, inequities still exist as low- and middle-income countries (LMIC) account for approximately 99% (302,000) of the global maternal deaths, with sub-Saharan Africa accounting for approximately 66% (201,000) of these deaths [2]. Economic and ethnic disparities are also evident in poorer countries based on the progress reports for the Millennium Development Goals [3]. Maternal mortality reduction remains a priority under the Sustainable Development Goal 3.1 with a target of less than 70 deaths per 100,000 live births by 2030 [2].

The majority of maternal deaths are preventable in LMICs, as 75% of all maternal deaths are caused by postpartum hemorrhage, hypertensive disorders of pregnancy (pre-eclampsia/eclampsia), infections, unsafe abortions and other delivery-related complications [4, 5]. High maternal mortality rates in LMIC have been associated with poor access to quality healthcare services during the antenatal, delivery and postnatal periods [6]. Accessible and quality antenatal care (ANC) and skilled birth attendance (SBA) during delivery have been shown to improve the survival and health outcomes of women in sub-Saharan Africa and Southeast Asia [7]. Evidence from studies has shown than 16–33% of maternal deaths can be averted with SBA at the time of delivery [4, 8–10].

In a recent systematic review of studies in LMICs, deliveries conducted within health facilities resulted in a 29% reduction in neonatal mortality; however, these results were found only within a conducive environment with skilled staff and emergency obstetrical facilities [11]. Several studies have shown that health facility-based deliveries may not be realistic for women living in rural and remote areas of LMIC due to poor physical access, long distances to facilities, and poor quality of services [11]. Furthermore, large proportions of unskilled deliveries still occur within health facilities [12]. Therefore, ensuring SBA at delivery, as opposed to facility-based deliveries, may be more appropriate to achieve when designing interventions to improve maternal outcomes for rural communities.

Several individual and contextual factors influence SBA during delivery. These include maternal age, parity, socio-economic status, education, cultural beliefs, access to quality and affordable care, and overall trust in the local healthcare system [13–16]. There is strong evidence that health promotion provided by community health workers (CHW) within the household, behavior change communication campaigns, early recognition of obstetrical complications, and prompt referral to higher levels of care can reduce delays in care-seeking and promote SBA during delivery [17]. CHWs perform a wide range of health promotion activities during home visits. These include treatment support, home-based care, promotion and facilitation of ANC attendance, use of culturally-acceptable educational strategies, engagement of family members in pregnancy-related care, and planning for a facility delivery [18]. However, few studies have explored the effect of various components of CHW service delivery on maternal care-seeking practices.

As a supportive mechanism for CHW systems, social accountability mechanisms using community scorecards have been integrated to improve health service utilization, including maternal and child health services in LMIC contexts [19–22]. The activities are focused to strengthen community engagement and people centered care by mobilizing communities and facility-oriented accountability mechanisms with health providers to improve service utilization and quality of care.

World Vision, a Christian relief and development organization, has made substantial investments in community-based health globally for maternal and child health in LMIC. These projects are implemented through comprehensive Area Development Programs and cover a wide range of services, including safe water and sanitation, health and nutrition education, child protection, food security and livelihood improvements. A multi-country mixed methods research study was conducted to determine the combined interventions of targeted CHW services and community oriented social accountability mechanisms using community scorecards on maternal, newborn and child health and nutrition. This study examines the associations between the timed and targeted CHW services and SBA at delivery.

## Methods

The 5-year multi-country research study was conducted between 2012 and 2017 in Cambodia, Kenya, Guatemala and Zambia by the Johns Hopkins University, the National Institute of Public Health in Cambodia, the Institute of Nutrition of Central America and Panama in Guatemala, Moi University School of Public Health in Kenya, and the Institute of Economic and Social Research at the University of Zambia. This analysis does not include results from the Guatemala study sites.

### Study design

The research was designed as a two-arm quasi experimental study in between September 2013 and September 2017. In each country, four districts or sub-districts with a population ranging from 19,000 to 25,000 were selected. Two districts/sub districts in each country were assigned to the intervention arm and two matched to the comparison arm based on several population, demographic, and access factors (population size, migratory patterns, accessibility to health facilities, disease burden, the presence of other health and non-health developmental programs, maturity and capacity of the World Vision Area Development programs) (Table 1).

**Table 1:** Selected Study Sites in Each Country

Study Sites	Intervention		Comparison	
Cambodia	Chulkiri	Comapa	Prasath Balang	Tbeng Meanchey
Kenya	Karemo	Katito	Kegonga-Ntimaru	Magunga
Zambia	Luampa	Magoye	Choongo	Nyimba

All selected study sites received regular programming from World Vision in the areas of water and sanitation, child protection, livelihood and economic development, and education. The study intervention was designed for a period of 24–36 months to enhance maternal, newborn, and child health. In the intervention sites, two combined interventions were launched; 1. Existing CHWs (and those newly recruited under the Ministry of Health in Cambodia) received a multi-phased training with three modules to provide targeted household health promotion and behavior change counseling and services at strategic stages during pregnancy, delivery, post-partum, and the early childhood period. 2. Social accountability mechanisms using Community Voice and Action and Community Scorecards were established to foster community governance and accountability and support health facility operations. Additional details on the mechanisms for social accountability for World Vision’s Community Voice and Action and Community Scorecards can be found elsewhere [23]. In both the intervention and comparison sites, World Vision facilitated the formation of facility management committees and community councils or strengthened existing councils to support CHWs and their services, using the Global Fund’s Community Systems Strengthening Framework [24]. The comparison sites continued to receive health services from the local district, and other development organizations including routine government supported CHW services.

In Cambodia, CHWs comprised of government-recruited Village Health Support Groups. Those in the intervention sites were trained in the timed and targeted counseling interventions. To augment the CHW workforce, additional CHWs were recruited and trained under the government program. Another cadre of Mother Groups were also trained in Cambodia as a supportive system for the CHWs. In Kenya the community health volunteers recruited by the government were trained using a cascade training strategy. World Vision teams trained the government staff, who trained the volunteers in timed and targeted counseling in the intervention sites. It is important to mention that in Kenya, the government had a very structured CHW system, with recruiting guidelines, tasks, reporting and supervision support with community health supervisors in all study sites. In Zambia, a cadre of community-based volunteer groups, termed Safe Motherhood Action Groups, established by the government’s safe motherhood program, were trained in timed and targeted counseling. Hence CHW recruitment, initial training, hours of work, task expectations, households covered (50–150), incentive systems, supervision, etc. varied in different countries.

We performed a multi-stage sampling strategy to select communities as sampling units in proportion to their population size. Households meeting the eligibility criteria were randomly selected for the interviews from each sampling unit. One eligible woman aged 15–49 years who was pregnant or had delivered in the previous 2 years and one child younger than 5 years of age were selected randomly from each eligible household. Sample size estimates were based on expected increase in skilled birth attendance. A two-sided alpha of 0.05 and power of 0.80 was used to determine the required sample size, with adjustments for non-response rate (5%) and a design effect of 1.2.

Interviewers with household survey experience received training on survey field procedures, ethics and informed consent. Appropriate quality control measures were employed for translation and field testing of instruments, data collection, and participant confidentiality. Structured household surveys, modified from the Demographic Health Surveys, were administered to all heads of households to obtain socio-demographic, food security, water and sanitation, and wealth asset information. Eligible women, 15–49 years, who were pregnant or delivered in the 2 years preceding the survey were interviewed using the Women’s survey, modified from the Demographic Health Surveys to obtain information on reproductive history, care-seeking behaviors, and utilization of health services for maternal, child and newborn health. The English language version of the women’s survey is included as a supplementary file. The Johns Hopkins University and local country Institutional Review Board approved informed written consent was obtained from all study participants in Kenya and Zambia, and verbal consent in Cambodia.

Facility-based ANC was defined as pregnancy-related care at a government or private hospital or clinic. A composite ANC Services Index (based on the WHO recommendations) was computed with a score of 0–12, with equal weight for the individual ANC services the woman received [25]. The ANC Services Index included: two doses of tetanus toxoid vaccine, iron or folate pills, antimalarial medications (in accordance with country policies), pregnancy-related nutrition counseling, counseling about the importance of danger signs in pregnancy, information on where to access care for antenatal or obstetrical complications, HIV testing, counseling on prevention of mother-to-child transmission of HIV, weight and blood pressure measurements, and testing of urine and blood.

To assess the degree and quality of pregnancy-related care provided by CHWs, we determined the number of CHW visits a woman received from the time of conception to delivery for her most recent pregnancy and other CHW service delivery quality indicators (CHW being courteous and respectful, woman’s satisfaction with CHW care, use of counseling aids or illustrated storybooks, pregnancy-related CHW counseling at home, inclusion of influential family members in pregnancy-related discussions, provision of information on pregnancy complications, discussion of solutions for any pregnancy-related problems, assistance with access to ANC, and follow up visits if the woman was referred to or visited a health center during her pregnancy). SBA was defined as deliveries that occurred in the presence of a doctor, clinical officer, nurse or midwife.

Standard quality control procedures were employed to clean, verify and analyze data using STATA 14 [26]. A principle components analysis using 12 household assets (television, radio, bicycle, etc.) and household type (type of roof, drinking water source, type of sanitation, etc.) were used to construct wealth quintiles. A descriptive analysis was performed by computing frequencies across the Intervention and Comparison sites, and *t* tests and chi-squared tests were performed to determine differences between intervention and comparison sites. Univariate logistic regressions were constructed to determine factors associated with SBA for maternal deliveries. The presence of collinearity among the independent variables used in the regression models was tested. Analyses were conducted separately for each country as contextual factors would inherently vary among the countries. Final models providing estimates of odds ratios for SBA were adjusted for: mother’s age, education, parity, wealth quintile, treatment arm, receiving 4 or more facility-based ANC visits, and ANC index score. The analysis includes results from the final evaluation, as there were minor variations in the baseline instruments. In Cambodia, a total of 3037 women were enrolled in the final evaluation, and 3037 were included in the analysis on SBA delivery care. In Zambia, 1194 women were enrolled, and 1171 included in the analysis, with a 1.9% missingness. Kenya had the highest missingness, 10.3%, as 3128 women were enrolled and only 2805 were included in the analysis. Missing records were eliminated from the analysis.

## Results

### Sociodemographic characteristics

Table 2 provides selected sociodemographic characteristics of women 15–40 years who reported a delivery in the past 2 years; 3037 in Cambodia, 2805 in Kenya and 1171 women in Zambia. More than 85% of households were headed by a male across all sites in both Cambodia and Kenya. In Zambia, less than 50% of households were headed by males in the intervention sites, compared to 70% in the comparison sites. In all three countries, most of the women were 20–36 years of age. More than 80% of the women were married in Cambodia and Kenya, while 70 to 75% of the women were married in Zambia.

**Table 2:** Sociodemographic characteristics of study population by country

Characteristics	Cambodia *N* = 3037			Kenya *N* = 2805			Zambia *N* = 1171		
	I *N* = 1261	C *N* = 1776	*p* value	I *N* = 1590	C *N* = 1215	*p* value	I *N* = 634	C *N* = 537	*p* value
	n(%)	n(%)		n(%)	n(%)		n(%)	n(%)	
Male-headed household	1130 (89.8)	1584 (89.5)	0.815	1360 (86.4)	1076 (89.3)	0.022	288 (46.3)	368 (69.7)	< 0.001
Mean family size	5.1	4.8	< 0.001	4.9	4.8	0.021	5.3	5.2	0.173
Mother’s age (in years)									
15–19	49 (3.9)	81 (4.6)	0.359	140 (8.8)	100 (8.2)	0.588	97 (15.3)	86 (16)	0.738
20–36	1113 (88.3)	1536 (86.5)	0.144	1334 (83.9)	1040 (85.6)	0.214	455 (71.8)	394 (73.4)	0.540
37–49	99 (7.8)	159 (8.9)	0.279	116 (7.3)	75 (6.2)	0.237	82 (12.9)	57 (10.6)	0.219
Marital status									
Married	1239 (98.3)	1744 (98.2)	0.906	1321 (83.4)	1121 (92.6)	< 0.001	438 (69.4)	399 (74.3)	0.064
Single/divorced/widow-ed	22 (1.7)	32 (1.8)	0.906	263 (16.6)	90 (7.4)	< 0.001	193 (30.6)	138 (25.7)	0.064
Highest education									
No education	191 (15.4)	453 (25.8)	< 0.001	19 (1.3)	52 (4.8)	< 0.001	44 (8.3)	68 (15.7)	< 0.001
Primary	675 (54.3)	764 (43.5)	< 0.001	1004 (69.2)	791 (72.7)	0.050	268 (49.8)	221 (50.9)	0.676
Secondary or more	377 (30.3)	539 (30.7)	0.831	429 (29.5)	244 (22.5)	< 0.001	225 (41.9)	145 (33.4)	0.004
Parity									
Primiparous	504 (40.0)	674 (38.0)	0.262	422 (26.6)	337 (27.7)	0.205	161 (25.4)	179 (33.3)	0.006
Multiparous	756 (60.0)	1098 (62.0)	0.275	1160 (73.4)	871 (72.3)	0.210	465 (74.6)	336 (66.7)	< 0.001
Had prior miscarriage or stillbirth	264 (20.9)	454 (25.6)	0.003	63 (4.0)	65 (5.4)	0.165	40 (6.3)	24 (4.5)	0.165
Wealth quintile									
Poorest (<20th percentile)	201 (15.9)	636 (35.8)	< 0.001	238 (15)	282 (23.3)	< 0.001	167 (26.1)	89 (16.6)	< 0.001
Poor (20th–39th percentile)	156 (12.4)	357 (20.1)	< 0.001	314 (19.7)	284 (23.4)	0.021	104 (16.4)	106 (19.7)	0.141
Middle (40th–59th percentile)	277 (22)	353 (19.9)	0.164	284 (17.9)	229 (18.8)	0.505	120 (18.9)	115 (21.4)	0.292
Rich (60th–79th percentile)	331 (26.2)	255 (14.4)	< 0.001	316 (19.9)	234 (19.2)	0.684	160 (25.2)	125 (23.2)	0.436
Richest (80th–99th percentile)	296 (23.5)	175 (9.8)	< 0.001	438 (27.5)	186 (15.3)	< 0.001	83 (13.1)	102 (18.9)	0.006
Has health insurance	333 (26.4)	536 (30.2)	0.022	–	–	–	–	–	–

*I* intervention, *C* comparison

In all three countries, more than 70% of the women had completed at least primary education, although this proportion was higher in Kenya compared to the other two countries. Approximately one-fourth of the women had access to health insurance in Cambodia, but this was not reported for Kenya or Zambia. Significant differences were evident between comparison and intervention sites for the following variables; male headed households (Kenya and Zambia), mean family size (Cambodia and Kenya), marital status (Kenya), education (Cambodia, Kenya and Zambia), health insurance (Cambodia) and wealth quintile (Cambodia, Kenya and Zambia).

### Antenatal and delivery care

Characteristics of ANC and delivery care are shown in Table 3. The WHO standard of receiving at least 4 or more facility-based ANC visits was significantly higher in the intervention sites for Cambodia (81.2% vs 58%, *p* < 0.001) and Kenya (70.5% vs 62.7%, p < 0.001) but was significantly higher in the comparison sites in Zambia (59.6% vs 73.2%, p < 0.001). In Cambodia, the mean month of women’s first ANC visit was the first trimester, whereas women in Kenya and Zambia tended to access ANC during the second trimester.

**Table 3:** Characteristics of Antenatal Care and Skilled Birth Attendance of Study Population by Country

Maternal careseeking characteristics	Cambodia *N* = 3037			Kenya *N* = 2805			Zambia *N* = 1171		
	I *N* = 1261	C *N* = 1776	*p* value	I *N* = 1590	C *N* = 1215	*p* value	I *N* = 634	C *N* = 537	*p* value
	n(%)	n(%)		n(%)	n(%)		n(%)	n(%)	
Antenatal care (ANC)									
*Received ≥ 4 facility ANC visits*	997 (81.2)	949 (58)	< 0.001	1048 (70.5)	645 (62.7)	< 0.001	321 (59.6)	254 (73.2)	< 0.001
*Month of 1st ANC visit (mean)*	2.4	2.7	< 0.001	4.4	4.3	0.014	4.2	3.8	< 0.001
*1st trimester*	994 (81.4)	1187 (73.3)	< 0.001	419 (28.8)	302 (29.9)	0.595	165 (30.8)	177 (51.1)	< 0.001
*2nd trimester*	212 (17.4)	391 (24.2)	< 0.001	942 (64.7)	654 (64.8)	0.908	356 (66.4)	167 (48.3)	< 0.001
*3rd trimester*	15 (1.2)	40 (2.5)	0.013	95 (6.5)	54 (5.3)	0.210	15 (2.8)	2 (0.6)	0.007
ANC services received									
*ANC Services Index Score* ^*b*^	9.2	7.8	< 0.001	10.0	9.8	< 0.001	9.9	9.2	< 0.001
*2 tetanus toxoid vaccination*	533 (42.5)	715 (42.1)	0.826	420 (27.2)	249 (23.6)	0.043	83 (14.1)	95 (18.9)	0.034
*Iron and folic acid*	1242 (99.1)	1638 (96.4)	< 0.001	1436 (94.1)	776 (71.1)	< 0.001	567 (97.4)	474 (97.5)	0.006
*Antimalarials*	19 (1.5)	17 (1.0)	0.222	1094 (71)	783 (74.4)	0.053	568 (96.4)	480 (95.8)	0.595
*Weight measurement*	1172 (93.7)	1468 (90.1)	< 0.001	1506 (99.3)	938 (90.2)	< 0.001	577 (99.5)	396 (99)	< 0.001
*Blood pressure measured*	1222 (97.5)	1481 (87.2)	< 0.001	1468 (96.8)	825 (79.3)	< 0.001	539 (92.9)	386 (96.5)	< 0.001
*Urine test*	671 (53.6)	396 (23.3)	< 0.001	1355 (89.4)	712 (68.4)	< 0.001	177 (30.5)	175 (43.8)	0.651
*Blood test*	1072 (85.6)	995 (58.6)	< 0.001	1327 (87.5)	664 (63.8)	< 0.001	560 (96.6)	383 (95.6)	< 0.001
*Nutrition advice*	1184 (94.7)	1466 (90)	< 0.001	1240 (81.6)	809 (78.6)	0.063	552 (95.3)	366 (92)	0.038
*Counseling on pregnancy complications and danger signs*	1144 (91.3)	1359 (80.4)	< 0.001	1331 (86.6)	813 (77.1)	< 0.001	546 (93.5)	451 (91.5)	0.215
*Informed about where to go if pregnancy-related complications develop*	1133 (99.1)	1341 (98.1)	0.019	1328 (99.5)	796 (98.2)	0.006	548 (99.3)	453 (99.1)	0.790
*Informed about PMTCT*^*c*^	1048 (84)	1260 (74.7)	< 0.001	1511 (98.1)	1004 (95.8)	0.002	560 (94.4)	490 (98)	0.002
*Offered HIV test*	1061 (84.7)	1139 (67.0)	< 0.001	1522 (98.7)	1033 (98.1)	0.318	591 (100)	491 (98.4)	0.083
Delivery care									
*Skilled birth attendance*	1249 (99.1)	1505 (84.8)	< 0.001	1460 (92.8)	1082 (91.7)	0.348	593 (94.1)	510 (95.3)	0.288
*Doctor*	157 (12.5)	143 (8.1)	< 0.001	241 (15.3)	215 (18.2)	0.043	14 (2.2)	48 (9.0)	< 0.001
*Clinical officer*	0 (0)	1 (0.06)	0.317	245 (15.6)	103 (8.7)	< 0.001	23 (3.6)	66 (12.4)	< 0.001
*Nurse*	3 (0.24)	6 (0.33)	0.608	900 (57.2)	714 (60.7)	0.073	490 (77.8)	280 (52.2)	< 0.001
*Midwife*	1089 (86.4)	1355 (76.3)	< 0.001	74 (4.7)	50 (4.2)	0.563	66 (10.5)	116 (21.7)	< 0.001
*Unskilled birth attendance*	12 (0.9)	271 (15.2)	< 0.001	122 (7.2)	107 (8.3)	0.348	41 (5.9)	27 (4.7)	0.288
*Community health worker*	0 (0)	20 (1.1)	< 0.001	24 (1.5)	10 (0.8)	0.098	6 (0.9)	5 (1.0)	0.978
*Relative/friend/neighbor*	1 (0.08)	5 (0.28)	0.174	34 (2.2)	40 (3.5)	0.055	19 (3)	8 (1.6)	0.078
*Traditional birth assistant (TBA)*	9 (0.7)	235 (13.3)	< 0.001	5 (0.3)	9 (0.8)	0.125	9 (1.5)	10 (1.9)	0.556
*No one*	1 (0.08)	11 (0.62)	0.008	50 (3.2)	37 (3.1)	0.955	3 (0.5)	1 (0.2)	0.385

*I* intervention, *C* comparison

a. Facility-based ANC includes: Private or Government facility, clinic, or hospital

b. ANC Services Index calculated by summing the total number of services (out of 12) each woman received during her last pregnancy, giving equal weight to each of the 12 services. Minimum score = 0; Maximum score = 12

c. PMTCT: prevention of mother to child transmission of HIV

A composite ANC services index score (maximum score = 12) was computed based on the quality of ANC services women received. The average score in all sites in all countries was above 9, except in the comparison site in Cambodia, where the average ANC index score was 7.8. In each country, women had significantly higher ANC services index scores in the intervention compared to the comparison sites. Table 3 shows the various individual ANC services received by women in each country, with significant differences between intervention and comparison sites for some services.

SBA during delivery was significantly higher in the intervention sites in Cambodia (99.1% vs 84.8, *p* < 0.001). While over 90% of women reported SBA during delivery in Kenya and Zambia, there were no significant differences between the intervention and comparison sites. In Cambodia, most women reported SBA from a midwife, while in Kenya and Zambia, majority of women received SBA from a nurse. Among the women who did not have SBA during delivery, the majority had a traditional birth assistant at delivery for Cambodia, whereas in Kenya and Zambia, the majority of those without SBA at delivery obtained care from a relative, friend or neighbor.

### Community health worker services

In all three countries, a significantly greater proportion of women in the intervention sites compared to comparison sites received at least one visit from a CHW during their last pregnancy (Table 4). The mean number of CHW visits varied between countries. In Zambia, more than 75% of women did not receive any visit from a CHW (76% in intervention sites and 89.9% in comparison sites, *p* < 0.001), while in Cambodia, 70% of the women in intervention sites reported at least 1–2 CHW visits, and 65.5% reported no CHW visits in the comparison sites. In all three countries regardless of treatment site, less than one-fourth of women received 3–4 CHW visits, and less than 5% of women received more than 4 CHW visits during their last pregnancy, except for the intervention sites in Kenya (10.5%). Across all countries, about one third of the CHW visits occurred during the 1st trimester.

**Table 4:** Components of community health worker (CHW) visits and services by country

	Cambodia			Kenya			Zambia		
Reported CHW Services	I *N* = 1261	C *N* = 1776	*p* value	I *N* = 1590	C *N* = 1215	*p* value	I *N* = 634	C *N* = 537	*p* value
	n(%)	n(%)		n(%)	n(%)		n(%)	n(%)	
CHW visited during last pregnancy	896 (71.8)	613 (35)	< 0.001	864 (55.1)	367 (31)	< 0.001	157 (24.8)	59 (11)	< 0.001
Mean Number of CHW visits	2.4	2.5	0.081	3.5	2.9	< 0.001	2.5	2.9	0.131
None	364 (29.2)	1146 (65.5)	< 0.001	769 (49.1)	835 (70.5)	< 0.001	477 (76)	478 (89.9)	< 0.001
1–2 visits	526 (42.1)	352 (20.1)	< 0.001	259 (16.6)	164 (13.9)	< 0.001	76 (12)	25 (4.7)	< 0.001
3–4 visits	321 (25.0)	202 (11.5)	< 0.001	373 (23.8)	144 (12.2)	< 0.001	69 (11)	22 (4.1)	< 0.001
> 4 visits	46 (3.7)	50 (2.9)	< 0.001	164 (10.5)	40 (3.4)	< 0.001	6 (1.0)	7 (1.3)	< 0.001
Mean month of 1st CHW visit	4.6	4.1	< 0.001	4.4	5.0	< 0.001	4.9	4.5	0.213
1st trimester	295 (33.9)	277 (46.6)	< 0.001	305 (35.4)	81 (22.1)	< 0.001	31 (20.1)	19 (33.9)	0.057
2nd trimester	422 (48.6)	233 (39.2)	< 0.001	446 (51.6)	214 (58.3)	0.032	97 (63)	26 (46.4)	0.036
3rd trimester	152 (17.5)	85 (14.2)	0.097	112 (13.0)	72 (19.6)	0.127	26 (16.9)	11 (19.7)	0.419
CHW was courteous and respectful	888 (99.1)	602 (98.7)	0.453	847 (98.4)	358 (98.4)	0.978	143 (96.6)	48 (96)	0.845
Satisfied with CHW services	884 (99)	601 (98.4)	0.305	844 (97.9)	339 (93.6)	0.002	141 (95.9)	45 (90)	0.202
CHW provided counseling during home visits	843 (94.9)	418 (69.8)	< 0.001	813 (97.7)	339 (97.4)	0.762	144 (99.3)	47 (88.7)	0.020
CHW used counseling aids/ storybooks	703 (79)	153 (25.2)	< 0.001	766 (89.1)	259 (71.2)	< 0.001	136 (90.1)	34 (64.2)	< 0.001
CHW discussed pregnancy complications	692 (77.8)	339 (55.6)	< 0.001	813 (94.4)	322 (88.7)	0.002	137 (91.3)	28 (54.9)	< 0.001
CHW provided solutions and recommendations for woman’s concerns	625 (70.2)	336 (55.1)	< 0.001	801 (93.3)	317 (87.8)	0.007	136 (90.1)	34 (69.4)	0.005
CHW included influential family members in pregnancy-related discussions	614 (69.6)	342 (56.6)	< 0.001	734 (85.7)	244 (67.4)	< 0.001	138 (91.4)	29 (56.9)	< 0.001
CHW facilitated woman’s access to ANC	551 (92.8)	218 (89)	0.097	711 (95.7)	270 (92.8)	0.086	82 (54.3)	22 (84.6)	0.751
CHW conducted a follow-up visit after referral or visit to a health center	600 (77.2)	194 (59.1)	< 0.001	429 (90.7)	260 (89.7)	0.641	68 (72.3)	17 (48.6)	0.018

*I* intervention, *C* comparison

Over 95% of women in all sites reported that the CHW was courteous and respectful and were satisfied with the CHW services. There were no significant differences between intervention and comparison sites, except in Kenya in terms of CHW service satisfaction, where women from intervention sites reported slightly higher levels of satisfaction (97.9% vs 93.6%, *p* = 0.002). In all three countries, most women reported that CHWs provided counseling during home visits with significant differences between intervention and comparison sites for Cambodia (94.9% vs 69.8%, *p* < 0.001) and Zambia (99.3% vs. 88.7%, *p* < 0.02). CHWs in intervention sites were significantly more likely to use counseling aids and storybooks during their visits, discuss pregnancy complications and danger signs with the woman, and include influential family members in the discussions compared to comparison sites in all three countries. For Cambodia and Kenya, over 90% of women reported that CHWs facilitated their access to ANC, in contrast to Zambia (53.3% in intervention sites and 84.6% in comparison sites). Approximately 90% of the women in the intervention and comparison sites in Kenya reported that CHWs made a follow-up visit after they were referred to or visited a health center, whereas in Cambodia and Zambia, it was lower, 75 and 50% in the intervention and comparison sites.

### Regression analysis

Table 5 shows results from the univariate and multivariate logistic regression analysis. Controlling for other factors, women 24 years or older had significantly greater odds (aOR = 1.65; 95% CI: 1.14, 2.39, *p* < 0.01) of receiving SBA in Cambodia. There was no significant association between age and SBA in Kenya and Zambia. Women with primary or secondary education had greater odds of receiving SBA compared to women with no education in Cambodia. Multiparous women compared to primiparous women and those in the wealth quintile equal to or above the 40th percentile compared to women whose wealth was less than the 40th percentile, who were otherwise similar on controlled factors, were significantly more likely to receive SBA. Women in the intervention sites in Cambodia had 7.5 times greater odds (aOR = 7.48; 95% CI: 3.87, 14.5) of receiving SBA compared to women in the comparison sites. The reverse was true for Kenya, where women had lower odds of having SBA at delivery in the intervention sites compared to the comparison sites (aOR = 0.60; 95% CI: 0.41, 0.85). There was no significant association between the study sites for SBA in Zambia.

**Table 5:** Multivariate Logistic Regression of Factors Associated with Skilled Birth Attendance by Country

Characteristics	Cambodia				Kenya				Zambia			
	SLR		MR		SLR		MR		SLR		MR	
	OR	95%CI	aOR	95%CI	OR	95%CI	aOR	95%CI	OR	95%CI	aOR	95%CI
Age												
< 24 yrs. (ref)	Ref	–	Ref	–	Ref	–	Ref	–	Ref	–	Ref	–
> = 24 yrs	1.10	0.84, 1.45	1.65 **	1.14, 2.39	0.80	0.60, 1.08	0.94	0.65, 1.36	0.48 *	0.28, 0.84	0.75	0.32, 1.77
Education												
None (ref)	Ref	–	Ref	–	Ref	–	Ref	–	Ref	–	Ref	–
Primary	2.88 ***	2.19, 3.79	1.96 ***	1.44, 2.69	1.64	0.85, 3.19	1.37	0.63, 2.97	1.78	0.86, 3.70	1.46	0.60, 3.53
Secondary +	5.02 ***	3.48, 7.24	2.96 ***	1.95, 4.47	5.38 ***	2.49, 11.63	3.61 **	1.48, 8.79	1.8	0.84, 3.85	1.31	0.49, 3.50
Parity												
Primi (ref)	Ref	–	Ref	–	Ref	–	Ref	–	Ref	–	Ref	–
Multi	2.23 ***	1.68, 2.97	2.31 ***	1.59, 3.34	1.78 ***	1.25, 2.52	1.49	0.96, 2.32	2.20 *	1.14, 4.27	2.21	0.78, 6.26
Wealth												
< 40th percentile (ref)	Ref	–	Ref	–	Ref	–	Ref	–	Ref	–	Ref	–
> = 40th percentile	4.70 ***	3.52, 6.26	2.12 ***	1.53, 2.94	1.38 *	1.05, 1.81	1.16	0.84, 1.59	1.06	0.65, 1.75	1.08	0.57, 2.03
Study Arm												
Comparison (ref)	Ref	–	Ref	–	Ref	–	Ref	–	Ref	–	Ref	–
Intervention	18.74 ***	10.5, 33.6	7.48 ***	3.87, 14.5	1.18	0.90, 1.55	0.60 **	0.41, 0.85	0.76	0.46, 1.26	0.82	0.42, 1.59
Facility-based ANC												
< 4 visits (ref)	Ref	–	Ref	–	Ref	–	Ref	–	Ref	–	Ref	–
> = 4 visits	4.48 ***	3.52, 5.96	1.66 **	1.20, 2.29	2.88 ***	2.18, 3.80	1.91 ***	1.36, 2.67	2.40 ***	1.43, 4.00	2.53 **	1.33, 4.81
ANC Index^a^ (1–12)	1.30 ***	1.25, 1.35	1.13 ***	1.08, 1.19	1.21 ***	1.17, 1.26	1.19 ***	1.13, 1.24	1.16 **	1.06, 1.28	1.08	0.94, 1.23
**CHW Visits and Services**												
Number of CHW visits	1.39 ***	1.25, 1.55	0.96	0.82, 1.12	1.08 *	1.01, 1.17	1.06	0.92, 1.21	0.93	0.77, 1.11	0.73	0.44, 1.20
CHW was courteous	2.70 ***	2.06, 3.54	2.91	0.32, 26.7	1.22 *	1.01, 1.76	3.00	0.75, 12.1	0.74	0.40, 1.36	5.19	0.17, 15.8
Satisfied with CHW	2.65 ***	2.02, 3.47	0.31	0.03, 2.96	1.28	0.97, 1.69	0.99	0.19, 5.13	0.73	0.40, 1.34	0.27	0.01, 8.18
CHW provided counseling during home visits	3.23 ***	2.38, 4.38	1.06	0.61, 1.86	1.25	0.94, 1.67	0.95	0.29, 3.31	0.80	0.43, 1.50	0.65	0.04, 10.1
CHW used counseling aids/story books	5.77 ***	3.63, 9.15	1.58	0.79, 3.17	1.24	0.93, 1.66	0.95	0.41, 2.19	0.78	0.41, 1.48	0.26	0.03, 2.63
CHW discussed pregnancy complications	3.57 ***	2.52, 5.05	1.10	0.51, 2.35	1.23	0.93, 1.63	0.66	0.16, 2.71	0.84	0.43, 1.63	0.17	0.01, 3.46
CHW provided solutions to concerns	2.98 ***	2.12, 4.18	0.65	0.30, 1.41	1.23	0.93, 1.63	0.28	0.06, 1.32	0.98	0.49, 1.96	10.5	0.72, 15.3
CHW included influential family in discussions	3.25 ***	2.29, 4.62	1.69	0.92, 3.13	1.52 **	1.12, 2.06	2.12 *	1.06, 4.26	0.96	0.48, 1.92	6.78 *	1.15,13.9
CHW facilitated access to ANC	3.23 ***	2.18, 4.78	0.66	0.37, 1.18	1.29	0.96, 1.74	0.70	0.33, 1.49	1.01	0.42, 2.39	4.01	0.68, 24.8
CHW follow up after referral /facility visit	6.58 ***	3.94, 10.97	2.44 ***	1.30, 4.60	1.85 ***	1.28, 2.67	2.17 **	1.25, 3.75	0.56	0.26, 1.22	1.89 *	1.05, 2.02

*SLR* simple logistic regression, *MLR* multivariate logistic regression, *OR* odds ratio, *aOR* adjusted odds ratio

ANC Index calculated by summing the total number of services (out of 12) each woman received during her last pregnancy, giving equal weight to each of the 12 services. Minimum score = 0; Maximum score = 12

*p < 0.05; **p < 0.01; ***p < 0.001

The odds of SBA were significantly higher for women who received four or more facility-based ANC visits for all three countries controlling for other factors. For Cambodia and Kenya, for every additional ANC service received (i.e., a one-point increase in the ANC Index Score), women were 1.13 times (95% CI: 1.08, 1.19) and 1.19 times (95% CI: 1.13, 1.24) more likely to receive SBA.

Though there was a significant positive dose-effect between the number of CHW visits and delivery with SBA in the univariate analysis for Cambodia and Kenya, this significance was no longer evident in the multivariate analysis. Controlling for other factors, women in Kenya and Zambia had a greater odds of SBA at delivery if the CHW included influential family members in discussions (Kenya aOR = 2.12; 95% CI: 1.06, 4.26; Zambia, aOR = 6.78; 95% CI: 1.15, 13.9), and in all three countries, if the CHW conducted a follow up visit after a referral to a health care center (Cambodia, aOR = 2.44; 95% CI: 1.30, 4.60; Kenya, aOR = 2.17; 95% CI 1.25, 3.75; Zambia, aOR = 1.89; 95% CI: 1.05, 2.02). Other components of CHW pregnancy-related services were not significantly associated with presence of SBA at delivery in the multivariate analyses.

## Discussion

It is postulated that more than 80% of maternal deaths can be prevented with supervision by a skilled professional at delivery [9, 27]. The findings from this study provide some evidence of the effectiveness of integrated community interventions focused on timed and targeted CHW health promotion to promote appropriate care-seeking in all study sites. Reported SBA was significantly higher for the intervention sites only for Cambodia, though over 90% reported SBA during delivery in both intervention and comparison sites for Kenya and Zambia. The type of SBA varied by country, mostly midwives and nurses in Kenya and Zambia. Other women sought care from traditional birth attendants in Cambodia and from friends, neighbors or relatives in Kenya and Zambia. It is apparent that the importance of SBA during delivery needs further emphasis in health promotion messaging during CHW household visits.

The key predictors of SBA, which varied across countries, were woman’s educational status, women aged 24 or more years, multiparity, higher wealth quintiles, four or more ANC visits, and number of ANC services, similar to findings reported in other studies [13–16]. CHW-related factors that showed a significant effect on likelihood of SBA were the inclusion of other family members in the decision making by CHWs and CHW follow up visits after referral or visit to the health facility. A study in Kenya on intent of SBA at ANC, also showed similar predictors; cost, educational level, number of ANC visits, and provider gender were significantly associated with women’s intent to deliver with an SBA [16].

Several studies have shown strong associations between ANC visits and SBA [28–30]. ANC also ensures the maternal care continuum as women who obtain ANC are more likely to access facility services for delivery and post-natal care [31]. Our study showed similar results as the number and type of ANC visits were key predictors of SBA. Women with 4 or more ANC visits had significantly higher odds of delivery with SBA, and ANC service index was significantly associated with the interventions in both Cambodia and Kenya. The specific components of ANC services included in our ANC index were not fully examined in previous studies [28–30].

A selected review of studies showed that differences in the extreme wealth deciles (as opposed to quintiles) were much larger than between the quintiles [32], highlighting the need to target additional support and CHW visits to these economically vulnerable households. Our study showed economic inequities, as women from lower wealth quintiles reported lower presence of SBA during delivery. Another multi-country analysis indicated that absolute household income was a better predictor of SBA wealth indices [33]. Absolute income was not considered in our model and may provide a better understanding of care-seeking patterns for SBA.

Evidence from other studies has shown that CHW visits during pregnancy have been significantly associated with increased SBA [34–37]. In our study population across three countries, we observed that a significantly higher proportion of women in the intervention sites reported CHW visits than women in comparison sites. Comparing intervention sites across countries, a greater proportion of women in Cambodia received CHW visits (71.8%) compared to Kenya (55.1%) and Zambia, where only one quarter of women (24.8%) received at least one CHW visit. These differences are likely due to other contextual factors. In Zambia, the CHWs were volunteers who had been initially recruited and trained by the ministry as Safe Motherhood Action Groups. Evidence from qualitative findings from this study, which are not included here, demonstrated major challenges in the implementation of timed and targeted counseling by CHWs in Zambia, including the lack of transport to reach remote households. High CHW attrition rates and low levels of satisfaction among CHWs were also reported. In Cambodia, a national policy limiting two CHWs per village was a major barrier to care, as the CHWs felt overwhelmed to meet the demands and expectations for service delivery. Data from the CHW qualitative findings also showed that the support systems for CHW supervision and oversight were also suboptimal, which may have resulted in the lack of a dose-effect between the number of CHW visits and SBA.

Despite the expansion of primary health care systems, CHW activities and service delivery vary greatly across and even within countries [18, 38]. Location and frequency of CHW visits during the antenatal period, and the number and content of counseling messages delivered can vary even within large-scale integrated CHW programs [18, 39]. This is likely due to the lack of standardization and sparse data on optimizing CHW service delivery. One study conducted in Nigeria showed that there was a positive dose-effect on several maternal and child health indicators from the intensity of CHW services delivered, measured by the number of one-on-one advice and assistance sessions provided in addition to standard pregnancy-related education and counseling [40]. Other studies have also looked at the dose-effect of CHW services on maternal care seeking practices but focused mostly on CHW program intensity. Karim et al. illustrated that a composite measure of time spent with the woman and number of counseling messages delivered was associated with improvements in ANC, iron supplementation, birth preparedness measures, and postnatal care, but not with facility deliveries or SBA [41]. Though we found a dose effect between number of CHW visits and SBA in the univariate analysis for Cambodia and Kenya, these results were no longer significant when controlled for other confounders.

Incentivizing CHWs with direct compensations as the accredited ASHA program in India, where CHWs perform a wide range of services, may be considered in these rural contexts [42]. The management of these systems need to be carefully regulated, as they have shown that incentives are both empowering and a source of distress due to low incentive rates relative to work expectations, irregular and/or incomplete payment, and the tendency to focus on highly incentivized services rather than client priorities.

For Kenya and Zambia, women were significantly more likely to have SBA at delivery if the CHW had involved influential family members in discussions with the woman. While this finding was not significant in Cambodia in multivariate analysis, there was a positive association in the univariate analysis. Our findings are supported by studies from various countries that have demonstrated that a key component to establishing this trust was ensuring male involvement in the woman’s care [38, 43]. In Uganda, this was most pronounced in terms of birth preparedness, as men still dominate economic power and related decision making in many households [38]. However, when the husband was part of counselling during pregnancy, decision making around saving money to pay for delivery care and seeking care was perceived to be easier for the woman and family in general [38]. In Ethiopia, participation in family meetings was significantly associated with an increase in the reported completeness of maternal and newborn health care that women received during birth and the early postnatal period, even after controlling for sociodemographic characteristics and maternal and newborn use of health services [43]. Furthermore, this study also showed that women who had both antenatal care and family participation in their care were most likely to have SBA for delivery [43]. In many LMIC, especially in sub-Saharan Africa and Southeast Asia, women often do not have decision-making power with regard to health service utilization [16, 38, 44]. Thus, the involvement of other key household members, such as husbands and mothers-in-laws, may be beneficial for effective CHW health promotion.

The sociocultural environment was another critical factor for optimizing CHW functionality. In Cambodia, kinship, social hierarchical structures, religion, patron-client relations and collectivism were shown to impact the ability of CHWs to form relationships and influence decision-making for service utilization [45]. The CHW system in Cambodia fosters a strong identity with a structured induction training and support led by local government bodies that includes basic skills, such as communication and behavior change technique [45]. Furthermore, literature on the impact of CHW use of support tools demonstrates that having culturally appropriate tools, such as narratives available through videos and storybooks, can be an important and cost effective aid to CHWs, as illustrated in a study from Pakistan, where such tools facilitated dialogue between men and women to create greater awareness of maternal care [46]. This is important to understand because while our study did not show a significant association between the use of counseling aids and storybooks and SBA, this aspect of CHW services may still be paramount for their ultimate impact on maternal care.

Women were also significantly more likely to have SBA during delivery if a CHW had conducted one or more follow-up visits after a referral or visit the woman after a health center visit. This finding is supported by a study from Uganda where follow-up and feedback of mothers who had been referred to higher levels of care was perceived to be important in creating accountability for referral compliance and fostering confidence in the CHW service [38]. This enabled them to successfully complete the maternal and newborn care practices recommended by the CHW [38]. Another example is the Family Health Program in Brazil, which demonstrated how effective and formal integration of CHWs into the healthcare system can improve maternal and child care-seeking behaviors [43].

Most CHW studies involve interventions with many components of service delivery, making it difficult to isolate the individual effects of each component [44]. The lack of a statistically significant finding on health outcomes may be attributed to weak implementation fidelity and lack of process optimization of the individual components within the program design [44]. The mechanisms for CHW recruitment, training, management and support are central to the quality of services that they deliver.

Complimentary social accountability mechanisms using community scorecards combined with CHW services, have shown to enhance maternal and health outcomes in Malawi and India [8, 31, 47]. These strategies provide a forum to address social determinants of health and positively influence the utilization of healthcare services by creating effective accountability structures, fostering transparent dialogues with community entities and health providers at the primary care facilities, and enhancing performance of providers to deliver equitable quality of care. Though this study did not explore the effects of the integrated approach of CHW home visits and social accountability mechanisms independently, use of services improved significantly in the intervention sites for number of ANC services received, though SBA at delivery was only significant for Cambodia. Further investigation of the independent and combined effects of social accountability mechanisms and CHW services is warranted and can enhance and optimize CHW service delivery.

We report several potential study limitations. The quasi experimental cross-sectional design does not allow for causal inferences about the effectiveness of the integrated interventions on maternal care seeking practices. Recall bias on timing and number of CHW visits by the women may be another factor for bias in this study, as the woman’s recall was not corroborated with the CHW visit records. Thirdly, the dose effect of CHW visits can only be ascertained with additional data on the time spent during home visits, content covered and the quality of the visits; these data were not captured in our study. Certification or training of midwives was not evaluated in this study; thus, the inclusion of midwives in the definition of SBA may be considered another potential limitation. Finally, we acknowledge that the broad spectrum of health and development interventions, including the establishment or strengthening of community councils and ongoing CHW services in the comparison sites, also contributed to increased SBA utilization for delivery care. These effects were not independently examined with a difference in difference analysis.

## Conclusion

This study provides some evidence that community-oriented interventions which address the number and nature of CHW service delivery components for maternal and child health promotion can increase the likelihood of SBA during delivery. A standard minimum number of CHW visits, while also considering the unique sociocultural contexts of different LMICs, should be evidenced-based. CHW communication skills to build rapport and trust with mothers and families should be paramount in promoting appropriate care seeking. Complementary community-level interventions to enhance social accountability to ensure equitable access to and utilization of quality services at the primary care level need to be empirically explored in the future. Despite the limitations, the study findings provide some evidence that effective engagement of CHWs and social accountability mechanisms with community entities can enhance access to safe deliveries for women in these rural communities.

## Supplementary information

**Additional file 1.**

## Data Availability

Data sets used for analysis for the current research are not publicly available as the research was performed under a contractual agreement, but available from the corresponding author upon reasonable request.

## Abbreviations

CHW: Community Health Worker
SBA: Skilled Birth Attendance
ANC: Antenatal Care
