# Supplementary material for: Can people-centered community-oriented interventions improve skilled birth attendance? Evidence from a quasi-experimental study in rural communities of Cambodia, Kenya, and Zambia
Source: BMC Pregnancy Childbirth. 2020 Sep 5;20:514. doi: 10.1186/s12884-020-03223-0 (PMC7487807; doi:10.1186/s12884-020-03223-0)
Supplement: Supplementary file 1 — Additional file 1. [file 12884_2020_3223_MOESM1_ESM.pdf]

Individual Women's Survey (English) 2016

I. COVER PAGE

WOMEN MASTER ID (LL – NN – NNNN):.................................. ☐☐ - ☐☐ - ☐☐☐☐

Instructions to create a Master ID:

1. Indicate country: Cambodia (C), Guatemala (G), Kenya (K), and Zambia (Z) ***(1 Letter: C, G, K, Z)***
2. Indicate survey type: **H**ousehold (H), Individual **W**omen (W) Health **F**acility Assessment (F) or **N**ew Patient Sick Child Observation/Exit Interview (N) ***(1 Letter: H, W, F, or N)***
3. Indicate Area Development Program (ADP) code ***(2 digits, 01-04)***
4. Indicate identification number starting with 1000 ***(4 digits)***
5. L = Letter N=Number **Example: CH-01-1000** [This is the same line number as the Household Survey.]

Household Master ID (Copy from HH Survey): ☐☐ - ☐☐ - ☐☐☐☐

Household head name and line number: ________________________________ Line number : ☐☐

Province/County name: ________________________________ Code : ☐☐

District name: ________________________________ Code : ☐☐

Community Unit name (*Kenya only*): ________________________________ Code : ☐☐

Area Development Program (ADP) name: ________________________________ Code : ☐☐

Cluster/village name: ________________________________ Code : ☐☐

Name and line number of woman from household survey: __________________ Line number: ☐☐

**Interviewer visits**	**1**	**2**	**3**
**Date (MM/DD/YYYY)**			
**Interviewer name and code**	☐☐	☐☐	☐☐
**Result***	☐	☐	☐

***Result codes:**
1. Completed
2. Not at home
3. Refused
4. Partly completed
5. Incapacitated
6. Other (specify)________________

Start time: ☐☐:☐☐ **00:00-24:00**

End time: ☐☐:☐☐ **00:00-24:00**

<u>**ALL ELIGIBLITY QUESTIONS MUST BE ASKED AND RECORDED**</u>

Eligibility Screener *(Eligible women include women who are currently pregnant or have delivered during the previous two years)IF MORE THAN 1 ELIGIBLE WOMAN RANDOMLY SELECT ONE WOMAN*

1. Are you between the ages of 15-49 years?.. Yes ☐ No ☐
2. Are you currently pregnant?.. Yes ☐ No ☐
3. Have you delivered during the previous two years?... Yes ☐ No ☐
4. Are you a caretaker of children under five? .... Yes ☐ No ☐

*IF <u>Q1 **AND** (Q2 **OR** Q3) **AND** Q4</u> are checked "yes" proceed to Module 4, Q401*

*IF <u>**ONLY** Q1 **AND** Q4 OR Q4</u> proceed to Module 9, Q901. IF MORE THAN 1 CHILD UNDER FIVE YEARS, RANDOMLY SELECT ONE CHILD*

If proceeding directly to Module 9, is the respondent the biological mother of the child under 5?.................. ……………………………………………………………………………Yes ☐ No ☐

Language of survey**: ________________________________ Code : ☐☐

Language of interview**: ________________________________ Code : ☐☐

Primary language of respondent**: ________________________________ Code : ☐☐

Translator used? .... Yes ☐ No ☐

**Language codes:	4.Lozi	7.Swahili
1.English<br>2. Khmer<br>3. Kaqchiquel	5.Nyanja<br>6.Spanish	8.Tonga<br>9.Other(Specify)_________

Supervisor: ☐☐<br>Name: ____________________<br>Date: ____________________<br>Signature: ________________	Field Editor: ☐☐<br>Name: ____________________<br>Date: ____________________<br>Signature: ________________	Office Editor:<br>☐☐	Keyed By:<br>☐☐

Instructions to interviewers:
For ALL questions, read each question option, except "Don't know" ***unless otherwise instructed****. For your response, select only one option per question* ***unless otherwise instructed****. Do not read instructions in italics aloud.*

NO.	QUESTIONS AND FILTERS
**000**<br>**C**	Throughout the survey you will see the symbol next to a question. This refers to a ***STOP AND CHECK*** point for the survey and will require the surveyor to check on a previous question. ***DO NOT LEAVE THIS PART BLANK***. Surveyors must answer this question.

Module 4: Female Respondents Background and Reproductive History

NO.	QUESTIONS AND FILTERS	CODED CATEGORIES	SKIP
**401**	In what month and year were you born?	Month................................ |__|__| Year |__|__||__|__| Don't know................................98	
**402**	What is your current marital status?	Currently married........................ 1<br>Divorced .... 2<br>Separated.................................. 3<br>Widowed.................................... 4<br>Never married .... 5<br>Refusal 6	 *→404*<br>*→404*<br>*→404*<br>*→404*<br>*→404*
**403**	At what age did you get married?	Age in years....................... |__|__| Don't know................................98	
**404**	Have you ever given birth?	Yes .... 1<br>No.. 2	 *→412*
**405**	At what age did you give birth to your first live-born child (even if he/she died later)?	Age in years....................... |__|__| Don't know................................98	
**406**	Do you have any children to whom you have given birth who are now living with you?	Yes .... 1<br>No.. 2	 *→408*
**407**	A. How many sons live with you?<br>B. How many daughters live with you?	Sons at home .... |__|__| Daughters at home............ |__|__| *[If none, record 00]*	
**408**	Do you have children to whom you have given birth but who do not live with you?	Yes .... 1<br>No.. 2	 *→410*
**409**	A. How many sons live elsewhere?<br>B. How many daughters live elsewhere?	Sons elsewhere................. |__|__| Daughters elsewhere .... |__|__| *[If none, record 00]*	
**410**	Have you ever given birth to a child who was born alive but later died? *[Explain: A baby born alive is one that cried, moved, or breathed after birth, even if died only a few minutes later]*	Yes .... 1<br>No.. 2	 *→412*
**411**	A. How many sons died?<br>B. How many daughters died?	Sons who died................... |__| Daughters who died .... |__| *[If none, record 00]*	

NO.	QUESTIONS AND FILTERS	CODED CATEGORIES	SKIP
**412**	Women sometimes have pregnancies that do not result in a live-born child. That is, a pregnancy can end in a miscarriage, or the child can be born dead. Have you ever had a pregnancy that did not end in a live birth?	Yes .... 1<br>No 2	 →*414*
**413**	How many pregnancies have you had that did not end in a live birth?	Pregnancy losses .... |__|__| *[If none, record 00]*	
**414**<br>**C**	Sum answers to 407, 409, and 411	A. 407A + 407B .... |__|__| B. 409A + 409B .... |__|__| C. 411A + 411B .... |__|__| D. Total (A+B+C) .... |__|__| *[If none, record 00]*	
**415**<br>**C**	Just to make sure that I have this right: you have had in TOTAL ______ live births during your life. Is this correct?	Yes .... 1<br>No 2	
**416**<br>**C**	Check **414:**<br>One or more live births *[proceed to Module 5]*<br>No births *[skip to Module 6: 601]*	One or more live births .... 1<br><br>No births 2	→*M5*<br><br>→*M6*

Module 5: Birth History

Now I would like to record the names of all your live births, whether they are still alive or not, starting with the first one you had. RECORD NAMES OF ALL THE BIRTHS IN 501. RECORD TWINS AND TRIPLETS ON SEPARATE LINES. (IF THERE ARE MORE THAN 14 BIRTHS, USE AN ADDITIONAL QUESTIONNAIRE, STARTING FROM THE SECOND ROW).

501	502	503	504	505	506	507	508	509	510
What name was given to your first/ next baby?<br>*RECORD NAME*<br>*BIRTH HIS-TORY NUMBER*	Is (NAME) a boy or a girl?	Were any of these births twins?	In what month and year was (NAME) born?<br>*PROBE: What is his/her birthday?*	Is (NAME) still alive?	How old was (NAME) at his/her last birthday?<br>*RECORD AGE IN COM-PLETED YEARS.*	Is (NAME) living with you?	***IF ALIVE,*** *RECORD HOUSE-HOLD LINE NUMBER OF CHILD (RECORD '00' IF CHILD NOT LISTED IN HOUSE-HOLD).*	How old was (NAME) when he/she died?<br>*IF '1 YR', PROBE: How many months old was (NAME)? RECORD DAYS IF LESS THAN 1 MONTH; MONTHS IF LESS THAN TWO YEARS; OR YEARS IF GREATER THAN 2 YEARS OF AGE AT DEATH*	*Were there any other live births between (NAME OF PREVIOUS BIRTH) and (NAME), including any children who died after birth?*
01	BOY 1<br>GIRL 2	SING 1<br>MULT 2	MONTH ☐☐<br>YEAR ☐☐☐☐	YES 1<br>NO 2<br>*If NO, go to 509*	AGE IN YEARS ☐☐	YES 1<br>NO 2	LINE NUMBER ☐☐<br>*(NEXT BIRTH)*	DAYS 1 ☐☐<br>MONTHS 2 ☐☐<br>YEARS 3 ☐☐	
02	BOY 1<br>GIRL 2	SING 1<br>MULT 2	MONTH ☐☐<br>YEAR ☐☐☐☐	YES 1<br>NO 2<br>*If NO, go to 509*	AGE IN YEARS ☐☐	YES 1<br>NO 2	LINE NUMBER ☐☐<br>*(skip to 510)*	DAYS 1 ☐☐<br>MONTHS 2 ☐☐<br>YEARS 3 ☐☐	YES 1<br>[Add birth]<br>NO 2<br>[Next birth]
03	BOY 1<br>GIRL 2	SING 1<br>MULT 2	MONTH ☐☐<br>YEAR ☐☐☐☐	YES 1<br>NO 2<br>*If NO, go to 509*	AGE IN YEARS ☐☐	YES 1<br>NO 2	LINE NUMBER ☐☐<br>*(skip to 510)*	DAYS 1 ☐☐<br>MONTHS 2 ☐☐<br>YEARS 3 ☐☐	YES 1<br>[Add birth]<br>NO 2<br>[Next birth]
04	BOY 1<br>GIRL 2	SING 1<br>MULT 2	MONTH ☐☐<br>YEAR ☐☐☐☐	YES 1<br>NO 2<br>*If NO, go to 509*	AGE IN YEARS ☐☐	YES 1<br>NO 2	LINE NUMBER ☐☐<br>*(skip to 510)*	DAYS 1 ☐☐<br>MONTHS 2 ☐☐<br>YEARS 3 ☐☐	YES 1<br>[Add birth]<br>NO 2<br>[Next birth]
05	BOY 1<br>GIRL 2	SING 1<br>MULT 2	MONTH ☐☐<br>YEAR ☐☐☐☐	YES 1<br>NO 2<br>*If NO, go to 509*	AGE IN YEARS ☐☐	YES 1<br>NO 2	LINE NUMBER ☐☐<br>*(skip to 510)*	DAYS 1 ☐☐<br>MONTHS 2 ☐☐<br>YEARS 3 ☐☐	YES 1<br>[Add birth]<br>NO 2<br>[Next birth]
06	BOY 1<br>GIRL 2	SING 1<br>MULT 2	MONTH ☐☐<br>YEAR ☐☐☐☐	YES 1<br>NO 2<br>*If NO, go to 509*	AGE IN YEARS ☐☐	YES 1<br>NO 2	LINE NUMBER ☐☐<br>*(skip to 510)*	DAYS 1 ☐☐<br>MONTHS 2 ☐☐<br>YEARS 3 ☐☐	YES 1<br>[Add birth]<br>NO 2<br>[Next birth]
07	BOY 1<br>GIRL 2	SING 1<br>MULT 2	MONTH ☐☐<br>YEAR ☐☐☐☐	YES 1<br>NO 2<br>*If NO, go to 509*	AGE IN YEARS ☐☐	YES 1<br>NO 2	LINE NUMBER ☐☐<br>*(skip to 510)*	DAYS 1 ☐☐<br>MONTHS 2 ☐☐<br>YEARS 3 ☐☐	YES 1<br>[Add birth]<br>NO 2<br>[Next birth]

501	502	503	504	505	506	507	508	509	510
What name was given to your first/ next baby?<br><br>*RECORD NAME*<br><br>*BIRTH HIS-TORY NUMBER*	Is (NAME) a boy or a girl?	Were any of these births twins?	In what month and year was (NAME) born?<br><br>*PROBE: What is his/her birthday?*	Is (NAME) still alive?	How old was (NAME) at his/her last birthday?<br><br>*RECORD AGE IN COM-PLETED YEARS.*	Is (NAME) living with you?	*RECORD HOUSE-HOLD LINE NUMBER OF CHILD (RECORD '00' IF CHILD NOT LISTED IN HOUSE-HOLD).*	How old was (NAME) when he/she died?<br><br>*IF '1 YR', PROBE: How many months old was (NAME)? RECORD DAYS IF LESS THAN 1 MONTH; MONTHS IF LESS THAN TWO YEARS; OR YEARS IF GREATER THAN 2 YEARS OF AGE AT DEATH*	*Were there any other live births between (NAME OF PREVIOUS BIRTH) and (NAME), including any children who died after birth?*
08	BOY 1<br>GIRL 2	SING 1<br>MULT 2	MONTH<br>YEAR	YES 1<br>NO 2<br>*If NO, go to 509*	AGE IN YEARS	YES 1<br>NO 2	LINE NUMBER<br>*(skip to 510)*	DAYS 1<br>MONTHS 2<br>YEARS 3	YES 1 [Add birth]<br>NO 2 [Next birth]
09	BOY 1<br>GIRL 2	SING 1<br>MULT 2	MONTH<br>YEAR	YES 1<br>NO 2<br>*If NO, go to 509*	AGE IN YEARS	YES 1<br>NO 2	LINE NUMBER<br>*(skip to 510)*	DAYS 1<br>MONTHS 2<br>YEARS 3	YES 1 [Add birth]<br>NO 2 [Next birth]
10	BOY 1<br>GIRL 2	SING 1<br>MULT 2	MONTH<br>YEAR	YES 1<br>NO 2<br>*If NO, go to 509*	AGE IN YEARS	YES 1<br>NO 2	LINE NUMBER<br>*(skip to 510)*	DAYS 1<br>MONTHS 2<br>YEARS 3	YES 1 [Add birth]<br>NO 2 [Next birth]
11	BOY 1<br>GIRL 2	SING 1<br>MULT 2	MONTH<br>YEAR	YES 1<br>NO 2<br>*If NO, go to 509*	AGE IN YEARS	YES 1<br>NO 2	LINE NUMBER<br>*(skip to 510)*	DAYS 1<br>MONTHS 2<br>YEARS 3	YES 1 [Add birth]<br>NO 2 [Next birth]
12	BOY 1<br>GIRL 2	SING 1<br>MULT 2	MONTH<br>YEAR	YES 1<br>NO 2<br>*If NO, go to 509*	AGE IN YEARS	YES 1<br>NO 2	LINE NUMBER<br>*(skip to 510)*	DAYS 1<br>MONTHS 2<br>YEARS 3	YES 1 [Add birth]<br>NO 2 [Next birth]
13	BOY 1<br>GIRL 2	SING 1<br>MULT 2	MONTH<br>YEAR	YES 1<br>NO 2<br>*If NO, go to 509*	AGE IN YEARS	YES 1<br>NO 2	LINE NUMBER<br>*(skip to 510)*	DAYS 1<br>MONTHS 2<br>YEARS 3	YES 1 [Add birth]<br>NO 2 [Next birth]
14	BOY 1<br>GIRL 2	SING 1<br>MULT 2	MONTH<br>YEAR	YES 1<br>NO 2<br>*If NO, go to 509*	AGE IN YEARS	YES 1<br>NO 2	LINE NUMBER<br>*(skip to 510)*	DAYS 1<br>MONTHS 2<br>YEARS 3	YES 1 [Add birth]<br>NO 2 [Next birth]

Module 6: Family Planning

Now I would like to talk about family planning – the various ways or methods that a couple can use to delay or avoid pregnancy.

NO.	QUESTIONS AND FILTERS	CODED CATEGORIES AND SKIP	SKIP
**601**	Are you pregnant now?	Yes .... 1<br>No .... 2<br>Don't know 8	 →*603*<br>→*603*
**602**	How many months pregnant are you?<br>*[Record number of completed months.]*	Number of months .... |__|__|	
**603**	Have you ever heard of ways or methods that a couple can use to delay or avoid a pregnancy?	Yes .... 1<br>No .... 2<br>Don't know 8	 →*701*<br>→*701*
**604**	Which ways or methods have you heard about?<br><br>*[Do not read out. For methods not mentioned spontaneously, ask: have you ever heard of (method)? Then proceed to **604A – 604X**, reading the name of each method not previously mentioned.]*		
	A. Female sterilization	Yes .... 1<br>No 2	
	B. IUD	Yes .... 1<br>No 2	
	C. Pill	Yes .... 1<br>No 2	
	D. Injections	Yes .... 1<br>No 2	
	E. Condom	Yes .... 1<br>No 2	
	F. Lactational amenorrhea method	Yes .... 1<br>No 2	
	G. Periodic abstinence	Yes .... 1<br>No 2	
	H. Withdrawal	Yes .... 1<br>No 2	
	I. Implants	Yes .... 1<br>No 2	
	X. Other	Yes .... 1<br>No .... 2<br>(Specify) ______________ X	
**605**	Have you ever done something or used any method to delay or avoid getting pregnant?	Yes .... 1<br>No 2	 →*701*
**606**<br>**C**	Check **601** for pregnancy status, the next series of questions (**607** - **608**) are **ONLY FOR WOMEN NOT CURRENTLY PREGNANT.**	Yes .... 1<br>No 2	→*701*

NO.	QUESTIONS AND FILTERS	CODED CATEGORIES AND SKIP	SKIP
**607**	Are you currently doing something or using any method to delay or avoid getting pregnant?	Yes ..... 1<br>No 2	 →*701*
**608**	Which method are you using now?<br>*[Record all mentioned. If none are mentioned do not record anything.]*		
	A. Female sterilization	Yes ..... 1<br>No 2	
	B. IUD	Yes ..... 1<br>No 2	
	C. Pill	Yes ..... 1<br>No 2	
	D. Injections	Yes ..... 1<br>No 2	
	E. Condom	Yes ..... 1<br>No 2	
	F. Lactational amenorrhea method	Yes ..... 1<br>No 2	
	G. Periodic abstinence	Yes ..... 1<br>No 2	
	H. Withdrawal	Yes ..... 1<br>No 2	
	I. Implants	Yes ..... 1<br>No 2	
	X. Other	Yes ..... 1<br>No ..... 2<br>(Specify) ____________ X	

Module 7: Antenatal, Delivery and Postnatal Care

Now, I would like to talk about your most recent pregnancy that resulted in a live birth.

NO.	QUESTIONS AND FILTERS	CODED CATEGORIES	SKIP
**701** **C**	Did you deliver in the previous two years?	Yes .... 1<br>No 2	 →*801*
**702** **C**	Did this pregnancy result in a live birth?	Yes .... 1<br>No .... 2<br>*[If NO, check if second-recent child was also delivered in the previous two years and resulted in a live birth, if none, skip to Module 8; 801]*	
**703** **C**	Check **501**: Record birth history number from **module 5** of most recent child delivered in the previous two years and resulted in a live birth.	Birth history number .... |__|__|	
**704**	Who makes the decision about whether to go for ANC?	Respondent .... 1<br>Husband/partner .... 2<br>Respondent and husband/ partner jointly .... 3<br>Other .... 4<br>(Specify) ________ 4A	
**705**	Did you consult anyone for antenatal care for this pregnancy with (NAME)?	Yes .... 1<br>No 2	 →*708*
**706**	Whom did you see for antenatal care for this pregnancy (NAME)?<br><br>Anyone else?<br><br>*[PROBE to identify each type of person and record all mentioned]*	Doctor .... A<br>Clinical officer .... B<br>Nurse .... C<br>Midwife .... D<br>Community Health Worker .... E<br>Other .... F<br>(Specify) ________ X	
**707**	Where did you receive antenatal care for this pregnancy (NAME)?	Government clinic .... 1<br>Govt/Social security hospital .... 2<br>Private clinic/hospital .... 3<br>Health post .... 4<br>CHW's house .... 5<br>Home .... 6<br>Other .... 7<br>(Specify) ________ 7A	→*709*<br>→*709*<br>→*709*
**708**	Why didn't you seek antenatal care in a clinic or hospital?<br><br>*[Do **not** read out. Record all mentioned. **After answering 708, skip to 714**]*	Didn't think it was necessary .... A<br>Transport too expensive .... B<br>Too far, no transportation .... C<br>Services too expensive .... D<br>No female provider at facility .... E<br>Unfriendly staff .... F<br>Inconvenient service hours .... G<br>Religious belief .... H<br>No one to accompany .... I<br>Other .... J<br>(Specify) ________ X	 <br><br><br><br><br><br><br><br><br>→*714*

NO.	QUESTIONS AND FILTERS	CODED CATEGORIES	SKIP
**709**	How many months pregnant were you when you first received antenatal care for this pregnancy with (NAME)?	Number of months............ |__|__| Don't know...............................98	
**710**	How many times did you receive antenatal care during this pregnancy with (NAME)?	Number of times .... |__|__| Don't know...............................98	
**711**	How many months pregnant were you when you went for your last antenatal care visit for this pregnancy with (NAME)?	Number of months............ |__|__| Don't know...............................98	
**712**	As part of your antenatal care during this pregnancy with (NAME), were any of the following done at least once?<br><br>*[Read out loud. Record all mentioned.]*	Weight .... A<br>Blood pressure .... B<br>Urine sample .... C<br>Blood sample............................ D<br>None of the above .... E	
**713**	As part of your antenatal care, were you given any advice about what to eat or not eat while pregnant with (NAME)?	Yes .... 1<br>No .... 2<br>Don't know............................... 8	
**714**	During your pregnancy, how many meals did you eat every day on average?	Number of meals .... |__|__|	
**715**	Is this number of meals more than usual, less than usual, or same as usual?	More than usual........................ 1<br>Less than usual .... 2<br>Same as usual.......................... 3	
**716**	During any of your antenatal care visit(s), were you told about pregnancy warning/danger signs?	Yes .... 1<br>No .... 2<br>Don't know............................... 8	 *→718*<br>*→718*
**717**	Were you told where to go if you had any complications?	Yes .... 1<br>No .... 2<br>Don't know............................... 8	
**718**	Do you know any symptoms during pregnancy that indicate the need to seek immediate care?	Yes .... 1<br>No .... 2<br>Don't know............................... 8	 *→720*<br>*→720*
**719**	Can you name any signs or symptoms that indicate the need for you to seek urgent care?<br><br>*[Do not read out. Record all mentioned]*	Fever .... A<br>Bleeding................................... B<br>Swelling of body/hands/ face..... C<br>Headaches .... D<br>Other.. E<br>(Specify) ____________________X	
**720**	During the pregnancy with (NAME), were you given an injection in the arm to prevent the baby from getting tetanus, that is, convulsions after birth?	Yes .... 1<br>No .... 2<br>Don't know............................... 8	 *→722*<br>*→722*
**721**	During the pregnancy with (NAME), how many times did you get this tetanus injection?	Number of times .... |__|__| Don't know...............................98	
**722**	At any time before this pregnancy with (NAME), did you receive any tetanus injections, either to protect yourself or another baby?	Yes .... 1<br>No .... 2<br>Don't know............................... 8	 *→725*<br>*→725*
**723**	Before this pregnancy with (NAME), how many other times did you receive a tetanus injection?	Number of times .... |__|__| Don't know...............................98	
**724**	How many months ago did you receive your **last** tetanus injection?<br>*[PROBE by reminding of important events that might have taken place around that year or age of her child.]*	Number of months............ |__|__| Don't know...............................98	

NO.	QUESTIONS AND FILTERS	CODED CATEGORIES	SKIP
**725**	During this pregnancy, were you advised about when to start breastfeeding?	Yes .... 1<br>No 2	
**726a**	During this pregnancy, were you given or did you buy any iron/iron folate tablets like these?<br><br>*[Show photo of iron/folate tablets. Explain that these tablets are given to prevent or treat anemia or low iron.]*	Yes .... 1<br>No .... 2<br>Don't know 8	
**726b**	During this pregnancy, were you given or did you buy any iron syrup?	Yes .... 1<br>No .... 2<br>Don't know 8	
**727**	Did you take any iron/folate tablets during your pregnancy with (NAME)?	Yes .... 1<br>No 2	 →*731*
**728**	For how long did you take the iron/folate tablets?	Less than 1 month .... 1<br>One to two months .... 2<br>Two to three months .... 3<br>Three months or more 4	
**729**	On average, how often did you take these iron/folate tablets during this pregnancy?	Daily .... 1<br>Weekly .... 2<br>Not often .... 3<br>Other .... 4<br>(Specify) ________ 4A	
**730**	Where did you get the iron/folate tablets from?	**Public sector**<br>Govt/Social security hospital ..... A<br>Health center/clinic .... B<br>Village health worker/ City health promoters .... C<br>Mobile / outreach clinic .... D<br>Facility council .... E<br>Other public .... F<br>(Specify) ________ F1<br>**Private Medical Sector**<br>Private hospital .... G<br>Private clinic .... H<br>Pharmacy .... I<br>Other private medical .... J<br>(Specify) ________ J1	
**731**	During this pregnancy, did you take any medicine to keep you from getting malaria?	Yes .... 1<br>No 2	 →*733*
**732**	What medicines did you take?<br><br>*[Record all mentioned. If type of medicine is not determined, show photo of typical antimalarial medicines to respondent]*	SP/Fansidar .... A<br>Chloroquine .... B<br>Other .... C<br>(Specify) ________ X<br>Don't know .... D	
**733**	During any of your antenatal visits, did anyone talk to you about babies getting the AIDS virus from their mother?	Yes .... 1<br>No 2	
**734**	Were you offered a test for the AIDS virus as part of your antenatal care?	Yes .... 1<br>No 2	 →*737*
**735**	I don't want to know the results, but were you tested for the AIDS virus as part of your antenatal care?	Yes .... 1<br>No 2	
**736**	I don't want to know the results, but did you get the results of the test?	Yes .... 1<br>No 2	

NO.	QUESTIONS AND FILTERS	CODED CATEGORIES	SKIP
**737**	During this pregnancy, did you take any medicine for intestinal worms?	Yes .... 1<br>No 2	
**738a**	Does your household have any mosquito nets that can be used while sleeping?	Yes .... 1<br>No 2	 →*742*
**738b**	Is this mosquito net a long-lasting insecticide-treated net (LLIN)?	Yes .... 1<br>No 2	→*741*
**739**	Since you got the mosquito net, was it ever soaked or dipped in a liquid to kill or repel mosquitoes?	Yes .... 1<br>No 2	 →*741*
**740**	How many months ago was the net last soaked or dipped?	Months .... |__|__|	
**741**	Did you sleep under this net last night?	Yes .... 1<br>No 2	
**742**	During your pregnancy, were you visited by a CHW? [use local term for CHW (SMAG, Madres Guias, VHSG]	Yes .... 1<br>No .... 2<br>Don't know 8	 →*801*<br>→*801*
**743**	When you were pregnant with (NAME), which month did the CHW first visit you?	Month pregnant .... |__| Don't know 8	
**744**	Where did you receive counseling from the CHW? *[Select all that apply]*	At home .... A<br>Outside the home .... B	
**745**	During your pregnancy with (NAME), how many times did a CHW visit you?	Number of times .... |__|__| Don't know 98	
**746**	How many times has a CHW visited you since you had (NAME)?	Number of times .... |__|__| Don't know 98	
**747**	When was the last time a CHW visited?<br>(Example 3 days ago, circle A and record 3 days)	A. Day.......... |__|__| B. Week .... |__|__| C. Month .... |__|__| D. Year .... |__|__| Don't know 98	
**748**	Did the CHW use storybooks to communicate about women's or child health?	Yes .... 1<br>No 2	
**749**	Did the CHW discuss any difficulties you were having in following the recommended practices?	Yes .... 1<br>No 2	
**750 a**	Did the CHW try to help you find solutions to the problems you have identified?	Yes .... 1<br>No 2	
**750b**	Were your decisions about the recommended practices written down in a household handbook?	Yes .... 1<br>No 2	
**751**	Did the CHW encourage influential family members to participate in discussions?	Yes .... 1<br>No 2	
**752**	At any time did the CHW check you or (NAME) and ask about any health problems you or (NAME) experienced recently?	Yes .... 1<br>No 2	 →*754*
**753**	Did the CHW help you get access to treatment that you needed?	Yes .... 1<br>No 2	
**754**	If you were referred at any time by the CHW, did the CHW also return to visit you after you returned from the facility?	Yes .... 1<br>No .... 2<br>Not referred 3	
**755**	If you were referred at any time by the CHW, did you go to the referred facility?	Yes .... 1<br>No 2	

NO.	QUESTIONS AND FILTERS	CODED CATEGORIES	SKIP
		Not referred 3	
**756**	Was the CHW courteous and respectful?	Yes .... 1<br>No 2	
**757**	Were you satisfied with the CHW services?	Yes .... 1<br>No 2	

Module 8: Delivery, Immediate Maternal Postpartum and Neonatal Care, and Breastfeeding

Now, I would like to talk about (NAME), your most recent birth.

NO.	QUESTIONS AND FILTERS	CODED CATEGORIES	SKIP
**801**<br>**C**	Check **701**: Did you deliver in the previous two years?	Yes .... 1<br>No 2	 →*901*
**802**	Who was the **primary** person to assist you during your delivery in the past 2 years?	Doctor .... 1<br>Clinical officer .... 2<br>Nurse .... 3<br>Midwife .... 4<br>Community Health Worker .... 5<br>Relative/neighbor/friend .... 6<br>No one .... 7<br>Other........ 8<br>(Specify) ________ 8A	
**803**	Where did you deliver?	Government clinic........ 1<br>Govt/Social security hospital .... 2<br>Private clinic/hospital........ 3<br>Health post .... 4<br>CHW's house .... 5<br>Home .... 6<br>Other........ 7<br>(Specify) ________ 7A	→*805*<br>→*805*<br>→*805*
**804**	What was the reason for not delivering in a clinic or hospital, government or private?<br><br>*[Do not read out. Record all mentioned.]*	Didn't think it was necessary........ A<br>Transport too expensive........ B<br>Too far, no transportation........ C<br>Services too expensive .... D<br>No female provider at facility........ E<br>Unfriendly staff .... F<br>Inconvenient service hours........ G<br>Religious belief........ H<br>No one to accompany .... I<br>Other........ J<br>(Specify) ________ X	
**805**	Who made the decision about where you should deliver?<br><br>*[Do not read out. Record the primary decision maker.]*	Myself .... 1<br>Husband .... 2<br>My mother........ 3<br>Mother-in-law........ 4<br>Father-in-law........ 5<br>Friends/neighbors........ 6<br>CHW .... 7<br>Midwife .... 8<br>Other health care provider........ 9<br>Other........ 10<br>(Specify) ________ 10A	
**806**	Was the birth delivered by cesarean section? *[EXPLAIN: It's when a child is delivered through a surgery.]*	Yes .... 1<br>No 2	
**807**	Immediately after (NAME) was born, was s/he dried and wrapped in a warm cloth or blanket?	Yes .... 1<br>No .... 2<br>Don't know........ 8	
**808**	Immediately after (NAME) was born, was the child's cord kept clean and dry?	Yes .... 1<br>No .... 2<br>Don't know........ 8	

NO.	QUESTIONS AND FILTERS	CODED CATEGORIES	SKIP
**809**	I would like to talk to you about follow-ups on your health after delivery, for example, someone asking you questions about your health or examining you. After (NAME) was born, did any one follow-up on your health and your newborn right after delivery?	Yes .... 1<br>No .... 2<br>Don't know................................. 8	 →*814*<br>→*814*
**810**	How long after (NAME) was born, did anyone follow up on your health? *[If less than one day, record hours. If less than one week, record days.]*	A. Hours......................... |__|__| B. Days .... |__|__| C. Weeks .... |__|__|	
**811**	Who made the follow up visit for your health and (NAME's) health the first time?	Doctor .... 1<br>Clinical officer .... 2<br>Nurse .... 3<br>Midwife .... 4<br>Community Health Worker .... 5<br>No one .... 6<br>Other... 7<br>(Specify) __________________7A	
**812**	When did you receive postnatal care after the delivery?<br><br>*Choose only one answer.*	Within 24 hours .... 1<br>>24 to <48hours……………….1a<br>48-72 hours .... 2<br>7-14 days................................... 3<br>6 weeks .... 4<br>Don't know................................. 5<br>None of the above 6	
**813**	Where did this follow-up take place?<br><br>(Government clinic can mean Health center, health clinic or health facility)	Government clinic...................... 1<br>Govt/Social security hospital ..... 2<br>Private clinic/hospital................. 3<br>Health post .... 4<br>CHW's house .... 5<br>Home .... 6<br>Other... 7<br>(Specify) __________________7A	→*815*<br>→*815*<br>→*815*
**814**	What was the reason for not having a postnatal check up in a clinic or hospital after this delivery?<br><br>*[Do not read out. Record all mentioned.]*	Didn't think it was necessary..... A<br>Transport too expensive............ B<br>Too far, no transportation .... C<br>Services too expensive .... D<br>No female provider at facility..... E<br>Unfriendly staff .... F<br>Inconvenient service hours........ G<br>Religious belief .... H<br>No one to accompany .... I<br>Other... J<br>(Specify) __________________ X	
**815**	After the delivery, were you given or did you buy any iron tablets or iron syrup?	Yes .... 1<br>No .... 2<br>Don't know................................. 8	
**816**	After the birth did you receive a vitamin A dose (like this)? *[Show photo of vitamin A dose]*	Yes .... 1<br>No .... 2<br>Don't know................................. 8	 →*820*<br>→*820*
**817**	In the first 2 months after delivery did you receive a vitamin A dose?	Yes .... 1<br>No 2	

NO.	QUESTIONS AND FILTERS	CODED CATEGORIES	SKIP
		Don't know ... 8	
**818**	Who provided you with the vitamin A dose?	Doctor ... 1<br>Clinical officer ... 2<br>Nurse ... 3<br>Midwife ... 4<br>CHW ... 5<br>Pharmacist ... 6<br>Relative/friend/neighbor ... 7<br>Other ... 8<br>(Specify) ________________ 8A	
**819**	How many total doses did you receive in the first 6 months after delivery?	Number of doses ... |__|__| Don't know ... 98	
**820**	Has (NAME) ever been breastfeed?	Yes ... 1<br>No ... 2	 →*829*
**821**	How long after birth did you first put (NAME) to the breast?	Immediately ... 1<br>Less than one hour after birth ... 2<br>Within 24 hours of birth ... 3<br>Other ... 4	
**822**	In the first 3 days after delivery, was (NAME) given anything to drink besides your breast milk?	Yes ... 1<br>No ... 2	 →*824*
**823**	What was (NAME) given to drink before your milk began to flow regularly?<br><br>Anything else?<br><br>*[Do not read out. Record all mentioned. If none of the items from A to X are mentioned, mark breast milk only.]*	Milk (not breast milk) ... A<br>Plain water ... B<br>Sugar or glucose water ... C<br>Gripe water ... D<br>Sugar-salt-water solution ... E<br>Fruit juice ... F<br>Tea/infusions ... G<br>Honey ... H<br>Infant formula ... I<br>Breast milk only ... J<br>Other ... K<br>(Specify) _________________ X	
**824**<br><br>**C**	CHECK **505**: Is (NAME) still living?	Yes, living ... 1<br>No, died ... 2	 →*839*
**825**	Are you still breastfeeding (NAME)?	Yes ... 1<br>No ... 2	
**826**	For how many months did you breastfeed (NAME)?<br>*[Record in months. If less than one month, record '00']*	Months ... |__|__|	
**827**	At what age do you think children should start receiving foods and liquids in addition to breast milk?	Less than 4 months ... 1<br>Between 4 – 6 months ... 2<br>Greater than 6 months ... 3	
**828**	Was (NAME) breastfed yesterday during the day or at night?	Yes ... 1<br>No ... 2<br>Don't know ... 8	
**829**	Now I would like to ask you about some medicines and vitamins that are sometimes given to infants. Was (NAME) given any vitamin drops or other medicines as drops yesterday during the day or at night?	Yes ... 1<br>No ... 2<br>Don't know ... 8	

NO.	QUESTIONS AND FILTERS	CODED CATEGORIES			SKIP
**830**	Was (NAME) given [LOCAL NAME FOR ORS] yesterday during the day or night?	Yes .... 1<br>No .... 2<br>Don't know 8			
**831**	Now I would like to ask you about liquids or foods (NAME) had yesterday during the day or at night.<br><br>Did (NAME) drink/eat:	**Yes**	**No**	**DK**	
	A. Plain water?	1	2	8	
	B. Commercially produced infant formula?	1	2	8	
	C. Any fortified, commercially available infant and young child food" [e.g. Cerelac]?	1	2	8	
	D. Any (other) porridge or gruel?	1	2	8	
**832**	Now I would like to ask you about (other) liquids or foods that (NAME) may have had yesterday during the day or at night. I am interested in whether your child had the item even if it was combined with other foods. Did (NAME) drink/eat:	**Yes**	**No**	**DK**	
	A. Milk (tinned, powdered, or fresh animal milk)?	1	2	8	
	B. Cheese, yogurt, or other milk products?	1	2	8	
	C. Bread, rice, noodles, or other grain foods?	1	2	8	
	D. White potatoes, white yams, manioc, cassava, or any other foods made from roots?	1	2	8	
	E. Pumpkin, carrots, squash, or sweet potatoes that are yellow or orange inside?	1	2	8	
	F. Any dark green leafy vegetables?	1	2	8	
	G. Ripe mangoes, papayas or *[insert any other locally available vitamin a-rich fruits]*?	1	2	8	
	H. Foods made with red palm oil, palm nut, palm nut pulp sauce?	1	2	8	
	I. Any other fruits or vegetables like oranges, grapefruit or pineapple?	1	2	8	
	J. Eggs?	1	2	8	
	K. Liver, kidney, heart or other organ meats?	1	2	8	
	L. Any meat, such as beef, pork, lamb, goat, chicken, or duck?	1	2	8	
	M. Fresh or dried fish or shellfish?	1	2	8	
	N. Grubs, snails, insects, other small protein food?	1	2	8	
	O. Any foods made from beans, peas, lentils, or nuts?	1	2	8	
	P. Any oils, fats, or butter, or foods made with any of these?	1	2	8	
	Q. Tea or coffee?	1	2	8	
	R. Any other liquids?	1	2	8	
	S. Any sugary foods, such as chocolates, candy, sweets, pastries, cakes, or biscuits?	1	2	8	
	T. Any other solid or soft food?	1	2	8	
**833**	Did (NAME) eat any solid, semi-solid, or soft foods yesterday during the day or night? If yes, PROBE: What kind of solid, semi-solid, or soft foods did (NAME) eat? *[Fill out category in* ***832****]*	Yes .... 1<br>No .... 2<br>Don't know 8			

NO.	QUESTIONS AND FILTERS	CODED CATEGORIES	SKIP
**834**	How many times did (NAME) eat solid, semi-solid, or soft foods other than liquids yesterday during the day or at night?<br>*[We want to find out how many times the child ate enough to be full. Small snacks and feeds such as one or two bites of mother's/sister's food should not be counted. Liquids do not count. Do not include thin soups or broth, watery gruels, or other liquid.]*	Number of times .... |__|__| Don't know...............................98<br><br>*[If caregiver answers seven or more times, record "7."]*	
**835**	When do you wash your hands with soap/ash?<br><br>*[Do not prompt. Circle all mentioned.]*	Never .... A<br>Before food preparation............. B<br>After defecation .... C<br>After attending to a child who has defecated.................................. D<br>Before feeding children .... E<br>Other... F<br>(Specify) _________________X	
**836**	The last time (NAME) passed stools, what was done to dispose of the stools?	Child used toilet or latrine.......... 1<br>Put into toilet/latrine .... 2<br>Put into drain/ditch..................... 3<br>Thrown into garbage .... 4<br>Buried .... 5<br>Left in the open.......................... 6<br>Other... 9<br>(Specify) _________________9A	
**837**	Was (NAME) measured against a growth chart by health worker either at home or at a health facility during the last 3 months?	Yes .... 1<br>No .... 2<br>Don't know................................. 8	
**838**	Did (NAME) receive an iron table or syrup in the last in the last week?<br>*[Show common types of tablets/syrups]*	Yes .... 1<br>No .... 2<br>Don't know................................. 8	
**839**	Who usually makes decisions about major household purchases?	Respondent .... 1<br>Husband/partner........................ 2<br>Respondent and husband/ partner jointly............................ 3<br>Someone else .... 4<br>Other... 5<br>(Specify) _________________5A	
**840**	Who usually makes decision about healthcare for yourself?	Respondent .... 1<br>Husband/partner........................ 2<br>Respondent and husband/ partner jointly............................ 3<br>Other... 4<br>(Specify) _________________4A	
**841**	Who usually makes decisions about your child's healthcare?	Respondent .... 1<br>Husband/partner........................ 2<br>Respondent and husband/ partner jointly............................ 3<br>Someone else .... 4<br>Other... 5<br>(Specify) _________________5A	

Module 9: Child Illnesses and Immunization

**C**	***STOP AND CHECK***<br>***THIS MODULE IS TO BE ADMINISTERED TO THE CAREGIVER (USUALLY THE MOTHER) OF CHILDREN RECORDED IN THE HOUSEHOLD ROSTER AS LESS THAN FIVE YEARS OF AGE. IF THERE IS MORE THAN ONE ELIGIBLE CHILD UNDER FIVE YEARS, SELECT ONE RANDOMLY***

Now, I would like to talk about (NAME).

NO.	QUESTIONS AND FILTERS	CODED CATEGORIES
**901**<br>**C**	**VERIFICATION:**<br>1. Check the respondents name and line number from household survey.<br>2. Check that the respondent is the primary caregiver (which is usually the mother) of (NAME).<br>3. Define the relationship of the respondent to (NAME).	Name ___________________<br>Line number from household survey<br>.. |__|__| <br>Yes .... 1<br>No... 2<br><br>*[If the person you are speaking to is not that individual ask to speak with the correct respondent].*<br><br>Biological Mother....................... 1<br>Female Caretaker 2
**IF MODULE 4-8 IS COMPLETED PROCEED TO Q902 AND 903, IF NOT PROCEED TO Q 903A**		
**902**<br>**C**	Check **501**: Birth history number from Module 5<br>[IF MODULE 4-8 IS COMPLETED]	Birth history number<br>........................... |__|__|
**903**<br>**C**	Check from **501** to **505**<br>[IF MODULE 4-8 IS COMPLETED]	1) Name_______________<br>2) Is [Name] alive?<br>1. Living |__| 2. Dead |__|
903a<br>**C**	Check HH line number for child U5<br>[IF MODULE 4-8 IS NOT COMPLETED]	Name____________________<br>Line number from household survey|__|__|

904	Did (NAME) sleep under a mosquito net last night?	Yes .... 1<br>No.............................. 2
**905**	Do you have a child vaccination/welfare card with (NAME)'s vaccination?<br>If yes, may I see it?	Yes, seen .... 1<br>*[skip to 907]*<br>Yes, not seen .... 2<br>*[skip to 910]*<br>No card 3
**906**	Did you ever have a vaccination card for (NAME)?	Yes .... 1<br>No.............................. 2 *[If no, skip to 910]*

907. (1) Copy vaccination date for each vaccine from card

(2) Write '44'in "Day" column if card shows that a vaccination was given, but no date recorded

(3) If more than two vitamin A doses, record dates for most recent and second most recent doses

	DD	MM	YYYY
BCG			
Check Scar on BCG injected place	1= Yes	2= No	
Hep B Birth Dose			
OPV 0			
OPV 1			
OPV 2			
OPV 3			
IPV			
DPT-Hep B-Hib 1			
Congenital immune control of Tetanus (PAB)	1= Yes	2= No	
DPT-Hep B-Hib 2			
DPT-Hep B-Hib 3			
PCV 1			
PCV 2			
PCV 3			
MR1			
MR2			
JE			
Vit A1			
Vit A2			
Yellow fever			
Rotavirus			

	Questions	
**908**	Check **907,** Are all vaccines recorded?	Yes .... 1<br>No............................. 2 *[continue to 909]*
**909**	Has (NAME) received any vaccinations that are not on this card, including vaccinations received in a national immunization campaign?<br>*[Record "yes" only if respondent says BCG, OPV 0-3, DPT 1-3 and/or measles vaccine. Probe for vaccinations and write "66" in the corresponding day column in 907]*	Yes .... 1<br>No............................. 2<br>Don't know .... 8<br>*If "No" or "DK" skip to 918*
**910**	Did (NAME) ever receive any vaccinations to prevent him/her from getting diseases, including vaccinations received in a national immunization campaign?	Yes .... 1<br>No............................. 2<br>Don't know .... 8<br>*If "No" or "DK" skip to 919*
**911**	Please tell me if (NAME) received any of these vaccines:<br>A BCG vaccination against tuberculosis, that is, an injection in the arm or shoulder that usually causes a scar.	Yes ....1<br>No............................2<br>Don't know 8
**912**	Polio vaccine, that is, drops in the mouth?	Yes ....1<br>No............................2<br>Don't know ....8<br>*If "No" or "DK" skip to 915*
**913**	Was the first polio vaccine received in the first 2 weeks after birth or later?	First 2 weeks...........1<br>Later ....2<br>Don't know 8
**914**	How many times was the polio vaccine received?	Number of times.......|__| Don't know 8
**915**	A pentavalent vaccination, which is an injection in the thigh, sometimes at the same time as polio drops?	Yes ....1<br>No............................2<br>Don't know ....8<br>*If "No" or "DK" skip to 917*
**916**	How many times was a Pentavalent vaccination received?	Number of times.......|__| Don't know 8
**917**	A measles injection- that is, a shot in the right upper arm at the age of 9 months or older- to prevent him from getting measles?	Yes ....1<br>No............................2<br>Don't know 8
**918**	Were any of the vaccinations (NAME) received during the last two years as a part of national immunization campaign?	Yes ....1<br>No............................2<br>No vaccines in the last two years….3<br>Don't know 8
**919**	Has (NAME) ever received a Vit. A dose (like this)?<br>*[Show common types of ampules/ capsules/syrups]*	Yes ....1<br>No............................2<br>Don't know ....8<br>*If "No" or "DK" skip to 921*

	Questions	
**920**	Did (NAME) receive a vitamin A dose within the last six months?	Yes ....1<br>No............................2<br>Don't know 8
**921a**	In the last seven days, did (NAME) take iron pills, sprinkles with iron, or iron syrup (like these)? *Show common types of pills/sprinkles/syrups*	Yes ....1<br>No............................2<br>Don't know 8
**921b**	Was (NAME) given any medicine for intestinal worms in the last 6 months?	Mebendazole ....1<br>Other antihelminth treatment…………….2<br>No............................3<br>Don't know 8
**922**	Has (NAME) had diarrhea in the last 2 weeks?	Yes ....1<br>No............................2<br>Don't know ....8<br>*If "No" or "DK" skip to 936*
**923**	Was there any blood in the stools?	Yes ....1<br>No............................2<br>Don't know 8
**924**	Now I would like to know how much was (NAME) given to **drink** during diarrhea (including breastmilk).<br>Was he/she offered much less than usual, somewhat less, about the same, more than usual or nothing?	Much less ....1<br>Somewhat less.........2<br>About the same........3<br>Nothing to drink........4<br>More ....5<br>Don't know 8
**925**	How much was (NAME) offered to **eat** during this diarrheal illness? Was he/she much less than usual, somewhat less, about the same, more than usual, or nothing?	Much less ....1<br>Somewhat less.........2<br>About the same........3<br>Nothing to eat..........4<br>More ....5<br>Don't know 8
**926**	Did you seek advice or treatment for (NAME) from **<u>any</u>** source?	Yes ....1<br>No............................2<br>*[If no, skip to 930]*
**927**	Where did you seek advice or treatment **first?**<br>*[Do not read. Record only one. SS stands for social security.]*	Government clinic ....1<br>Govt/SS hospital ......2<br>Private clinic/hospital3<br>Health post ....4<br>CHW's house ....5<br>Pharmacy ....6<br>Other ....9<br>(Specify)_________9A
**928**	Did you seek advice or treatment **anywhere else**?<br><br>*[Do not read. Record only one. SS stands for social security.]*	No other place..........1<br>Government clinic ....2<br>Govt/SS hospital ......3<br>Private clinic/hospital4<br>Health post ....5<br>CHW's house ....6<br>Pharmacy ....7<br>Other 9

	Questions	
		(Specify)_________9A
**929**	How many days after diarrhea began did you **first** seek advice or treatment for (NAME)? *If same day record '00*	Days ....|__|__|
**930**	Does (NAME) still have diarrhea?	Yes ....1<br>No............................2<br>Don't know 8
**931**	Was he/she given any of the following to drink at any time since he/she started having diarrhea?	
	A. Fluid from a special packet called Oralite or ORS.	Yes ....1<br>No............................2<br>Don't know 8
	B. Another home-made liquid such as porridge, soup, yoghurt, coconut water, fresh fruit, juice, tea, milk, or rice water?	Yes ....1<br>No............................2<br>Don't know 8
	C. A home-made sugar-salt solution	Yes ....1<br>No............................2<br>Don't know 8
	D. Fruit, juice, tea, milk, or rice water	Yes ....1<br>No............................2<br>Don't know 8
**932**	Was anything else given to treat diarrhea?	Yes ....1<br>No............................2<br>Don't know ....8<br>*If "No" or "DK" skip to 936*
**933**	What else was given to treat diarrhea?<br>Anything else?<br><br>*[Record all treatments.]*	**Pill or syrup**<br>Antibiotic..................A<br>Antimotility medicine B<br>Zinc ....C<br>Other ....D<br>Unknown ....E<br><br>**Injection**<br>Antibiotic..................F<br>Non-antibiotic ....G<br>Unknown ....H<br><br>Intravenous fluids.....I<br>Home remedy ....J<br>Other ....K<br>(Specify)_________ X
**934**<br>**C**	Check 933:<br>Was zinc given?	Yes ....1<br>No............................2<br>Don't know ....8<br>*If "No" or "DK" skip to 936*
**935**	How many times was zinc given?	Number of doses.|__|__| Don't know 98
**936**	Has (NAME) been ill with fever any time in the last 2 weeks?	Yes ....1<br>No............................2

	Questions	
		Don't know 8
**937**	In the last 2 weeks, has (NAME) had illness with a cough?	Yes..........................1<br>No............................2<br>Don't know ....8<br>*If "No" or "DK" skip to 940*
**938**	When (NAME) had an illness with a cough did (NAME) breathe faster than usual with short, rapid breaths or have difficulty breathing?	Yes..........................1<br>No............................2<br>Don't know ....8<br>*If "No" or "DK" skip to 941*
**939**	Was the fast or difficult breathing due to a problem in the chest or due to a blocked and runny nose?	Chest only................1<br>Nose only.................2<br>Both..........................3<br>Don't know ....8<br>Other ....4<br>(Specify)__________ 4A
**940**<br>**C**	A. Check **936**: Had fever?<br><br>B. Check **937**: Had cough?	Yes..........................1<br>No............................2<br>Don't know ....8<br><br>Yes..........................1<br>No............................2<br>Don't know ....8<br><br>If YES to **either** A or B, go to **941**<br>(child had either a cough or a fever)<br><br>If NO or DK to **BOTH** A and B, go to **955**<br>(child has not had either a cough or fever)
**941**	Now I would like to know how much was (NAME) given to drink (including breastmilk) during the illness with a (fever/cough).<br><br>Was he/she given less than usual to drink, about the same amount, or more than usual to drink?<br><br>*IF LESS, PROBE: Was he/she given much less than usual to drink or somewhat less?*	Much less.................1<br>Somewhat less.........2<br>About the same........3<br>Nothing to drink........4<br>More.........................5<br>Don't know 8
**942**	How much was (NAME) offered to eat during this illness with a fever/cough? Was he/she much less than usual, somewhat less, about the same, more than usual, or nothing?	Much less.................1<br>Somewhat less.........2<br>About the same........3<br>Nothing to eat...........4<br>More.........................5<br>Don't know 8
**943**	Did you seek advice or treatment for the illness with fever/cough from any source?	Yes..........................1<br>No............................2<br>*[If no, skip to 947]*
**944**	Where did you seek advice or treatment **first?**<br>*[Do not read. Record only one. SS stands for social security.]*	Government clinic ....1<br>Govt/SS hospital ......2<br>Private clinic/hospital3

	Questions	
		Health post...............4<br>CHW's house...........5<br>Pharmacy.................6<br>Other........................7<br>(Specify)_________ 7A
**945**	Did you seek advice or treatment **anywhere else**?<br><br>*[Do not read. Record only one. SS stands for social security.]*	No other place..........1<br>Government clinic ....2<br>Govt/SS hospital ......3<br>Private clinic/hospital4<br>Health post...............5<br>CHW's house...........6<br>Pharmacy.................7<br>Other........................8<br>(Specify)_________ 8A
**946**	How many days after fever/cough began did you first seek advice or treatment for (NAME)? [*If same day record '00*]	Days....................|__|__|
**947**	Is (NAME) still sick with the fever/cough?	Fever only ....1<br>Cough only...............2<br>Both..........................3<br>Neither......................4<br>Don't know 8
**948**	At any time during the illness, did (NAME) take any medicines for the illness?	Yes...........................1<br>No.............................2<br>Don't know ....8<br><br>*If no or don't know, skip to Q.955*
**949**	What medicines did (NAME) take?<br>Any other medicines?<br><br>*[Record all mentioned.]*	**Antimalarial Medicines**<br>SP/Fansidar ....A<br>Chloroquine..............B<br>Amodiaquine............C<br>Al/Coartem ....D<br>Quinine.....................E<br>Other........................F<br>(Specify) ________ Fa<br><br>**Antibiotic Medicines**<br>Pill/syrup...................G<br>Injection....................H<br>Other........................X<br>(Specify) ________ Xa<br>Don't know ....Z
**950**<br>**C**	Check **949**:<br>Any code A-F circled?	Yes...........................1<br>No.............................2<br>[*If no, go to 955]*

	Questions	
**951**	How long after the first fever did (NAME) take this medicines?	Days ....|__|__| Don't know 98
**952**	For how many days did (NAME) take this medicines?	Days ....|__|__| Don't know 98
**953**	Was anything else done about (NAME)'s fever?	Yes ....1<br>No.............................2<br>Don't know ....8<br>*If "No" or "DK" skip to 955*
**954**	What else was done about (NAME)'s fever?<br><br>*[Select all that apply]*	Consulted traditional healer ....A<br>Gave warm sponging…B<br>Gave herbs ....C<br>Other ....D<br>(Specify)__________ X

END: Please thank the respondent for their time.
